# Distinct biological signature and modifiable risk factors underly the comorbidity between major depressive disorder and cardiovascular disease

**DOI:** 10.1101/2023.09.01.23294931

**Published:** 2023-09-01

**Authors:** Jacob Bergstedt, Joëlle A. Pasman, Ziyan Ma, Arvid Harder, Shuyang Yao, Nadine Parker, Jorien L. Treur, Dirk J.A. Smit, Oleksandr Frei, Alexey Shadrin, Joeri J. Meijsen, Qing Shen, Per Tornvall, Alfonso Buil, Thomas Werge, Jens Hjerling-Leffler, Thomas D. Als, Anders D. Børglum, Cathryn M. Lewis, Andrew M. McIntosh, Unnur A. Valdimarsdóttir, Ole A. Andreassen, Patrick F. Sullivan, Yi Lu, Fang Fang

**Affiliations:** 1 Unit of Integrative Epidemiology, Institute of Environmental Medicine, Karolinska Institutet, Stockholm, Sweden; 2 Department of Medical Epidemiology and Biostatistics, Karolinska Institutet, Stockholm, Sweden; 3 NORMENT Centre, Institute of Clinical Medicine, University of Oslo and Division of Mental Health and Addiction, Oslo University Hospital, Oslo, Norway; 4 Genetic Epidemiology, Department of Psychiatry, Amsterdam UMC, University of Amsterdam, Amsterdam, the Netherlands; 5 Institute of Biological Psychiatry, Mental Health Center Sct. Hans, Mental Health Services Copenhagen, Roskilde, Denmark; 6 Clinical Research Center for Mental Disorders, Shanghai Pudong New Area Mental Health Center, Tongji University School of Medicine, Shanghai, China; 7 Institute for Advanced Study, Tongji University, Shanghai, China; 8 Department of Clinical Science and Education Södersjukhuset, Karolinska Institutet, Stockholm, Sweden; 9 The Lundbeck Foundation Initiative for Integrative Psychiatric Research (iPSYCH), Copenhagen, Denmark; 10 Lundbeck Foundation GeoGenetics Centre, GLOBE Institute, University of Copenhagen, Copenhagen, Denmark; 11 Department of Clinical Medicine, Faculty of Health and Medical Sciences, University of Copenhagen, Copenhagen, Denmark; 12 Department Medical Biochemistry and Biophysics, Karolinska Institutet, 171 77 Stockholm, Sweden; 13 Department of Molecular Medicine (MOMA), Molecular Diagnostic Laboratory, Aarhus University Hospital, Aarhus, Denmark; 14 Department of Biomedicine, Aarhus University, Aarhus, Denmark; 15 The Lundbeck Foundation Initiative for Integrative Psychiatric Research, iPSYCH, Aarhus, Denmark; 16 Center for Genomics and Personalized Medicine, Aarhus, Denmark; 17 Social, Genetic and Developmental Psychiatry Centre, King’s College London, London, UK; 18 Department of Medical and Molecular Genetics, King’s College London, London, UK; 19 Centre for Clinical Brain Sciences, University of Edinburgh, Royal Edinburgh Hospital, Edinburgh, UK; 20 Centre for Genomics and Experimental Medicine, Univeristy of Edinburgh, Edinburgh, UK; 21 Centre of Public Health Sciences, Faculty of Medicine, School of Health Sciences, University of Iceland, Reykjavik, Iceland; 22 Department of Epidemiology, Harvard TH Chan School of Public Health, Harvard University, Boston, Massachusetts, USA; 23 K.G. Jebsen Centre for Neurodevelopmental disorders, University of Oslo and Oslo University Hospital, Oslo, Norway; 24 Departments of Genetics and Psychiatry, University of North Carolina at Chapel Hill, NC, USA

## Abstract

Major depressive disorder (MDD) and cardiovascular disease (CVD) are often comorbid, resulting in excess morbidity and mortality. Using genetic data, this study elucidates biological mechanisms, key risk factors, and causal pathways underlying the comorbidity. We show that CVDs share a large proportion of their genetic risk factors with MDD. Multivariate genome-wide association analysis of the shared genetic liability between MDD and CVD revealed seven novel loci and distinct patterns of tissue and brain cell-type enrichments, suggesting a role for the thalamus. Part of the genetic overlap was explained by shared inflammatory, metabolic, and psychosocial risk factors. Finally, we found support for causal effects of genetic liability to MDD on CVD risk, but not vice versa, and demonstrated that the causal effects are partly explained by metabolic and psychosocial factors. The distinct signature of MDD-CVD comorbidity aligns with the idea of an immunometabolic sub-type of MDD more strongly associated with CVD than overall MDD. In summary, we identify plausible biological mechanisms underlying MDD-CVD comorbidity, as well as key modifiable risk factors for prevention of CVD in individuals with MDD.

Major depressive disorder (MDD) and cardiovascular disease (CVD) are highly comorbid^1,2^. Several hypotheses might explain the observed comorbidity^2^. One explanation is that genetic risk factors for MDD and CVDs overlap. While observed genome-wide genetic correlations between MDD and CVD are modest^2–4^, this may be because local genetic correlations of opposing directions attenuate correlations on the genome-wide level, leading to an underestimation of the genetic overlap^3^. The large polygenicity of MDD^4^ might also mask subtypes with stronger genetic relationships to CVD.

The observed MDD-CVD comorbidity could also be due to non-genetic factors. Cardiovascular risk factors like systolic blood pressure, low-density lipoprotein cholesterol, body mass index (BMI), physical inactivity, type 2 diabetes, and smoking have all been associated with MDD^5–7^. Moreover, accumulating data show that psychosocial factors associated with MDD, like educational attainment, childhood maltreatment, loneliness, and sleep duration, are also important risk factors for CVD^8–11^.

One common mechanism underlying MDD, CVD, and as well we their shared risk factors could be chronic inflammation. Atherosclerosis, the accumulation of fibrofatty lesions in the arterial wall, is the main cause of CVD^12^. The build-up of atherosclerotic plaque is a long-term inflammatory process mediated by immune components in crosstalk with arterial wall cells^13^. Many lines of evidence also support a role for inflammation in MDD^14^. Excessive or long-term psychosocial stress promote the maturation and release of inflammatory cytokines like interleukin (IL)-6, which activate the central nervous system to produce behaviors related to MDD^15^. Importantly, low-grade inflammation, defined by high C-reactive protein levels, has been observed in more than a quarter of patients with depression^16^, suggesting the presence of an inflammatory subtype of MDD^17^, which might be especially strongly associated with CVD.

The full extent of the genetic overlap between MDD and CVD has not been explored. It remains unknown if the genetic overlap is associated with specific tissues or brain cell-types, or how this overlap relates to shared risk factors such as blood pressure, psychosocial traits, metabolic traits, and inflammation. Moreover, causal effects linking these traits are not fully understood ^18–21^.

Here, we dissect the genetic overlap between MDD and CVD by leveraging state-of-the-art genomic data and methods (Fig. 1). We used newly released summary statistics from a genome-wide association study (GWAS) of MDD involving more than 300,000 cases^4^, with substantially increased statistical power compared to previous GWASs. First, we assessed the pairwise genetic overlap between MDD and CVD on the genome-wide level, as well as on the level of local partitions of the genome and overlapping causal variants with MiXeR^22^ and LAVA^23^. Using these methods, we were able to consider the direction of the association at each locus in the genome, providing a more granular understanding of the genetic overlap between MDD and CVD. Second, we identified genetic variants and genes that contribute to the shared liability between MDD and CVD using Genomic Structural Equation Modeling (SEM)^24^. We mapped identified variants to brain cell-types using novel annotations based on single-cell RNA sequencing in post-mortem human brain^25^. Third, we assessed shared risk factors explaining the association between MDD and CVD. To do so, we evaluated the polygenic overlap between MDD and well-established risk factors. We then estimated genetic correlation between MDD and CVD adjusting for risk factors, and the genetic correlation between the shared genetic liability between MDD and CVD and risk factors. Finally, we used Mendelian Randomization (MR) to investigate putative causal effects and assess mediation through shared risk factors for MDD and CVD. In brief, this study leverages recent large-scale GWAS data and triangulates results from current genomic methods to elucidate etiological pathways underlying the comorbidity between MDD and CVD.

## Results

### Most genetic risk factors for CVD overlap with MDD

**Extended Data Figure 1.**
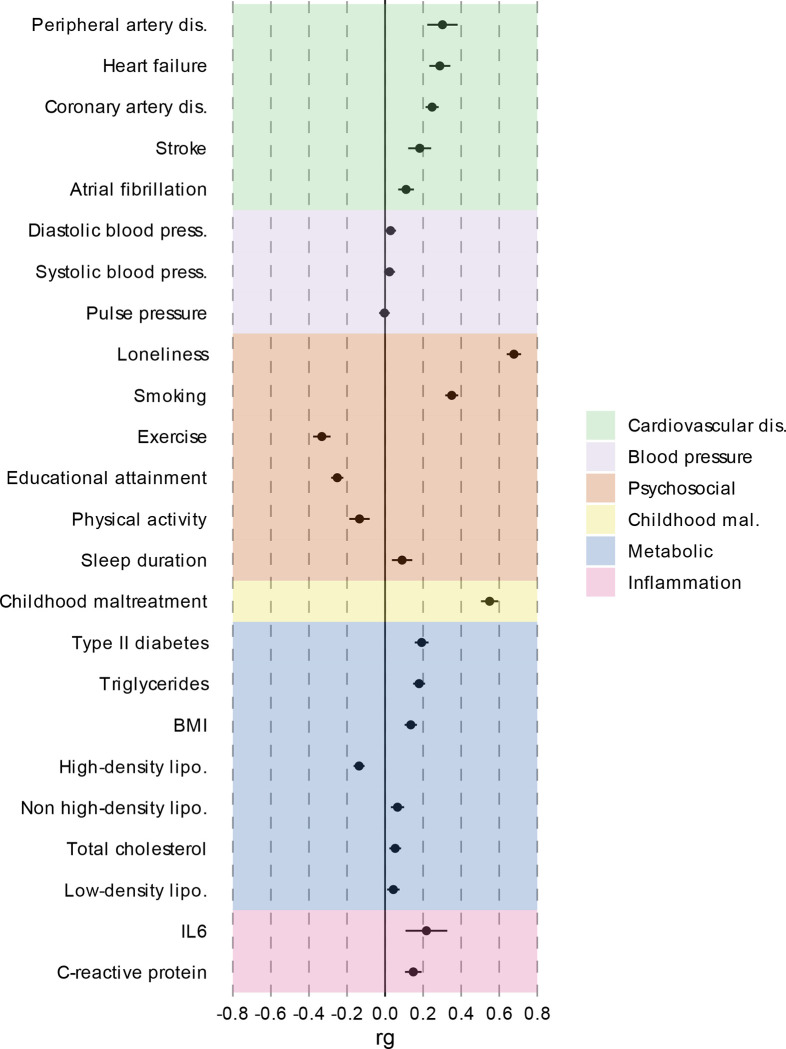
Genetic correlation between MDD and CVD and risk factors. Traits are color-coded using manual categorization. Bars represent 95% confidence intervals. Results are based on LD Score Regression analysis.

**Extended Data Figure 2.**
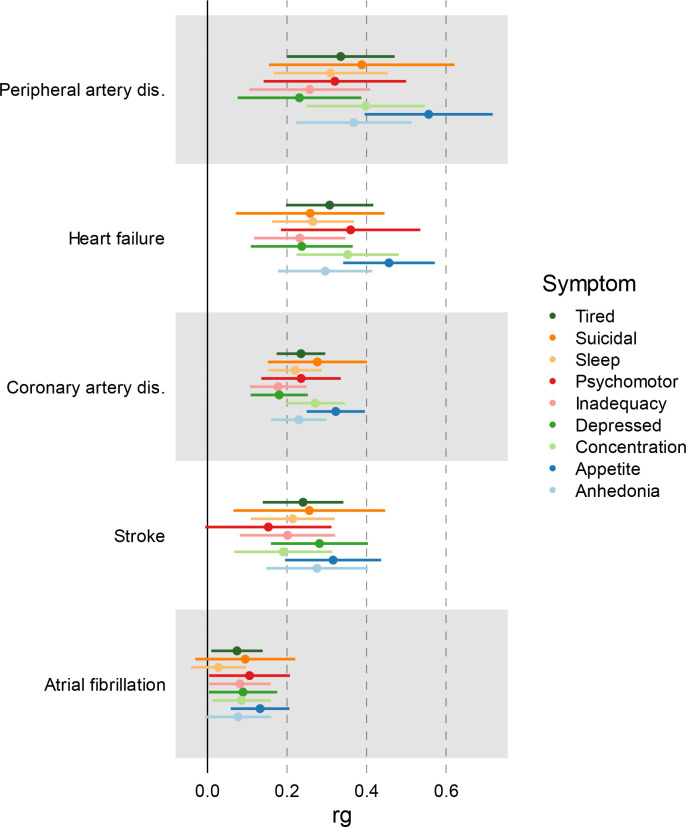
Genetic correlation between MDD symptoms and CVDs. Bars represent 95% confidence intervals. Results are based on LD Score Regression analysis.

There were weak to moderate genome-wide genetic correlations between MDD and CVDs (coronary artery disease, peripheral artery disease, heart failure, atrial fibrillation, and stroke), as estimated with linkage disequilibrium score regression (LDSC^26^; Extended Data Fig. 1, Table S1). The strongest correlations were noted for peripheral artery disease (*r*_*g*_=0.30, SE=0.04, *p*=1E-13), heart failure (*r*_*g*_=0.29, SE=0.03, *p*=1E-24), and coronary artery disease (*r*_*g*_=0.25, SE=0.02, *p*=9E-45), while smaller but statistically significant correlations were observed for stroke and atrial fibrillation (*r*_*g*_=0.18 and *r*_*g*_=0.11, *p*<1E-7). CVDs showed strong correlations among each other, except for atrial fibrillation, which was moderately genetically correlated to the other CVDs (Fig. S1, Table S1). To aid interpretation of these results, we also analyzed specific MDD symptoms. The genetic correlations between specific MDD symptoms (as listed in Table 1) and CVDs mirrored results for MDD diagnosis (Extended Data Fig. 2). Overall, changed appetite showed the strongest (average *r*_*g*_=0.36), whereas feelings of inadequacy showed the weakest (average *r*_*g*_=0.19), genetic correlations with CVDs (Table S2).

**Figure 2.**
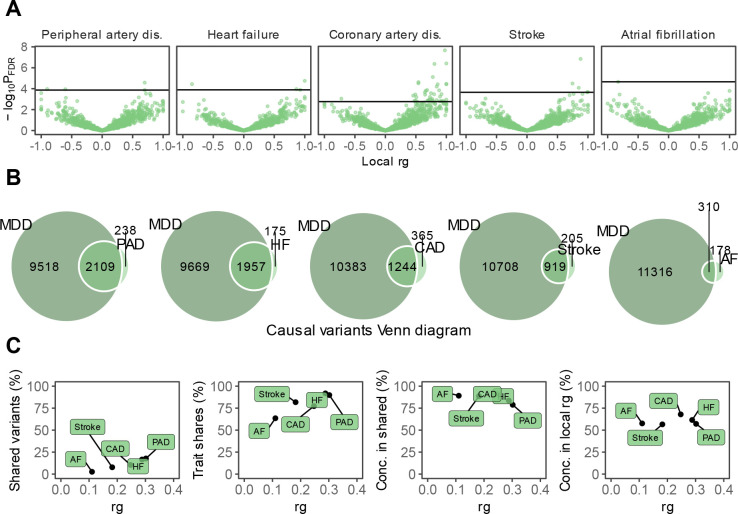
Genetic overlap between MDD and CVD beyond genome-wide genetic correlation. **A** Volcano plots based on LAVA results and shows the genomic loci (green dots, N=2,500) with the local genetic correlation between MDD and each of the CVDs (x-axis) and the corresponding p-value (y-axis). Loci exceeding the horizontal line are significant at *p*_FDR_<.05. **B** Venn diagrams based on MiXeR results and shows the number of causal variants that are unique to MDD (left circle), unique to CVD (non-overlapping part of right circle), or shared between MDD and CVD (overlapping part of circles). **C** Genetic correlation estimated by LDSC (rg, x-axis) against the percentage of MDD variants that are shared with the CVD trait as estimated by MiXeR (first plot), the percentage of CVD variants that are shared with MDD (second plot), and the percentage of CVD variants that are shared with MDD that have the same effect direction (third plot). The fourth plot shows the percentage of local genetic correlations from LAVA that are in the same effect direction on the y-axis. MDD=Major Depressive Disorder, PAD=Peripheral Artery Disease, CAD=Coronary Artery Disease, AF=Atrial Fibrillation, HF=Heart Failure

We then estimated local genetic correlations between MDD and CVDs in each of 2,500 distinct genomic regions used in LAVA^23^. The distribution of local genetic correlations is displayed in Fig. 2A, with points exceeding the horizontal line representing partitions with a genome-wide significant correlation (*p*_*FDR*_<0.05). We found 54 significant local correlations between MDD and CVDs, 40 of which were for MDD and coronary artery disease. Most local correlations were positive (90%), although results were mixed for peripheral artery disease and heart failure, with 50% and 25% of the partitions, respectively, showing negative correlations with MDD (Table S3).

Next, we investigated genetic overlap on the level of risk variants using MiXeR^22^ (Table S4). We identified more variants for MDD than for the CVDs, suggesting that MDD is more polygenic than CVD. Results showed that CVDs shared a large proportion of their causal variants with MDD (from 64% in atrial fibrillation to 92% in heart failure, Fig. 2B) whereas MDD shared only a small proportion of its causal variants with CVDs (<20%). Note that for peripheral artery disease the performance metrics of the model indicate that this finding needs to be interpreted with caution (Table S4). Both shared genetic variants and local genetic correlations exhibit strong degrees of effect direction concordance (Fig. 2C), suggesting that genetic risk variants for CVDs are strongly correlated with a genetic subcomponent of MDD.

### Shared genetic liability to MDD and CVD

**Figure 3.**
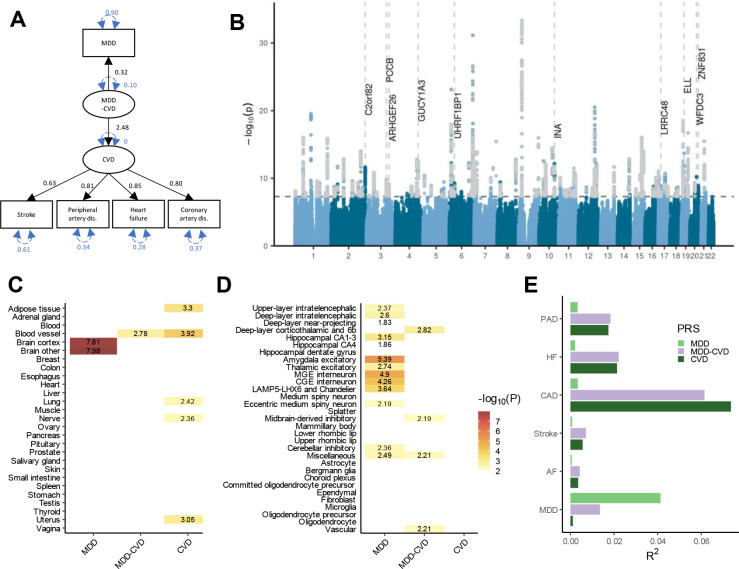
Shared genetic liability factor for MDD-CVD. **A** Latent factor model as specified in Genomic SEM with the ‘observed’ variables in rectangles and the latent variables in circles. Unstandardized factor loadings are given in black and variances in blue. **B** GWAS results on the latent MDD-CVD factor. The x-axis shows genomic position, and the y-axis shows statistical significance as −log_10_(*p*). Genome-wide significant SNPs with a significant heterogeneity Q that were filtered out (with a strong effect on one or some of the indicators that was not well explained through the common latent factor) are displayed in grey. The top 10 eQTL genes are displayed with the dashed vertical lines indicating their position. **C** Enrichment results in GTEx tissues for the latent MDD-CVD factor, with latent CVD (without MDD) and MDD-only as comparison. The exact log-transformed p-values are displayed on the tiles. Only tissues with a significant association (*p*_FDR_<.05) are shown. **D** Enrichment results for the latent MDD-CVD factor, latent CVD, and MDD-only in brain cell types. **E** Variance explained in MDD and CVD phenotypes in UK Biobank (defined using ICD-codes listed in Table S11) by each of 3 PRSs for the latent MDD-CVD factor, latent CVD, or MDD-only. MDD=Major Depressive Disorder; CVD-MDD=common factor for CVD and MDD; CVD=common factor for the cardiovascular diseases

To further characterize the genetic overlap, we explicitly modelled the shared liability between MDD and CVD as a higher-order latent factor using Genomic SEM. For this analysis, we excluded atrial fibrillation because it deteriorated the model fit and had a low factor loading. The final model had an excellent fit with Comparative Fit Index CFI>0.999 and Standardized Root Mean Squared Residual SRMR=0.021. The factor loading for MDD was *β*=0.32. The loadings for the CVDs ranged from *β*=0.63 for stroke (SE=0.06, *p*=4.37E-41) to *β*=0.85 for heart failure (SE=0.07, *p*=2.97E-56) (Fig. 3A). For comparison, we also fit a latent factor for CVD alone (without MDD), which showed similar fit and parameter estimates (CFI>0.999, SRMR=0.013) (Fig. S2). This shows that genetic liability to different CVDs as well as to CVDs and MDD (to a lesser extent) can be explained by a single underlying factor.

**Extended Data Figure 3.**
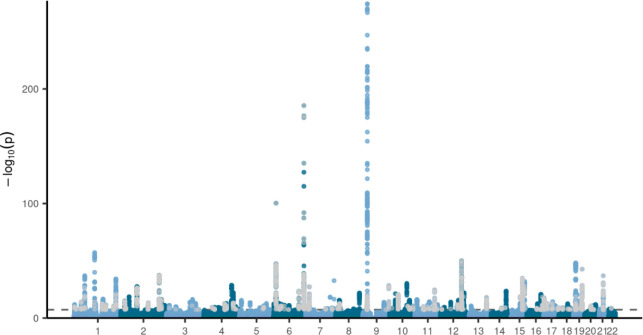
Manhattan plot of the GWAS results on the latent CVD factor (without MDD). Genome-wide significant SNPs with a significant heterogeneity Q (with a strong effect on one or some of the indicators that was not well explained through the common latent factor) are displayed in grey. The dashed line indicates the genome-wide significance threshold (p<5e-08).

**Extended Data Figure 4.**
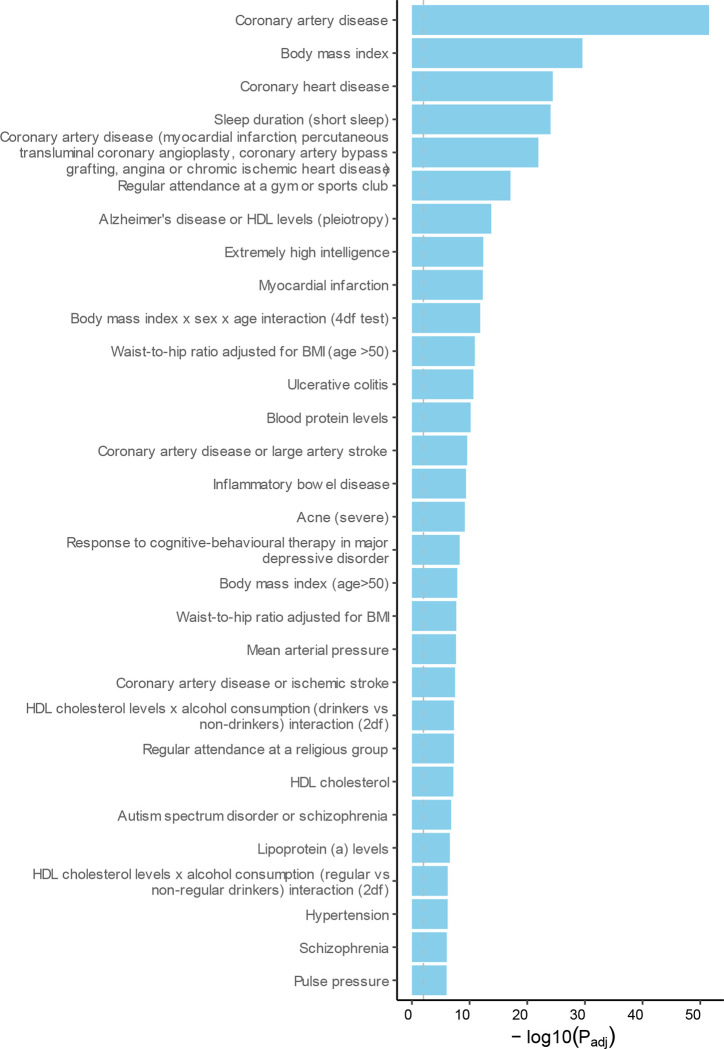
Enrichment for the MDD-CVD GWAS SNPs in other traits. Results from FUMA relying on the GWAS catalogue database. The traits are as reported in the original study. The dashed grey line indicates the significance threshold after FDR-adjustment.

**Extended Data Figure 5.**
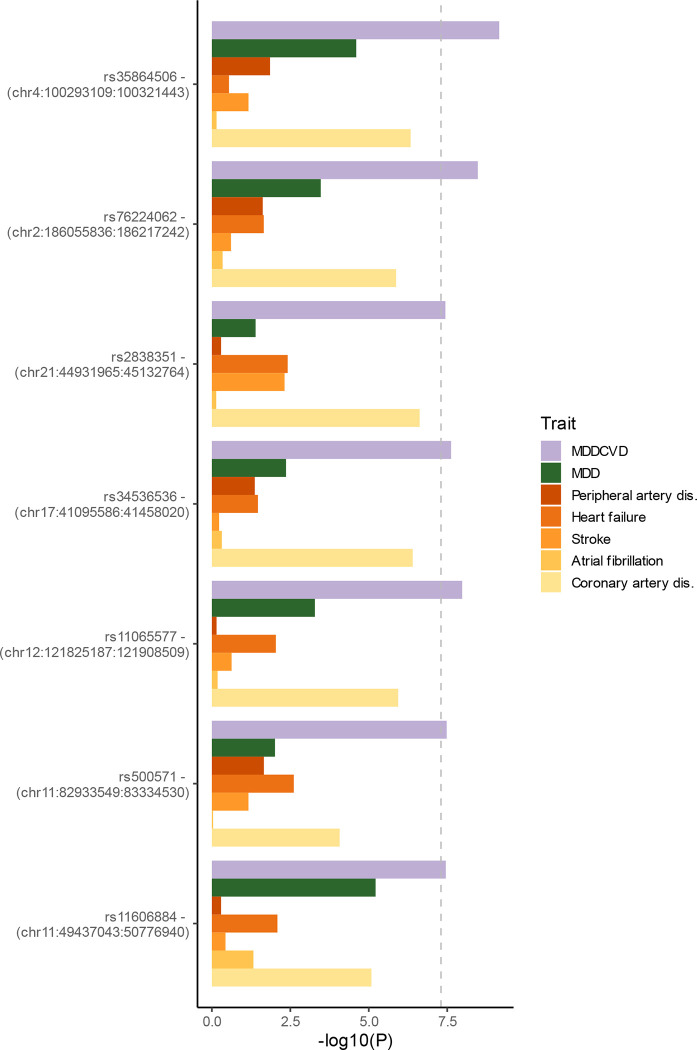
Unique loci for latent MDD-CVD. On the y-axis are SNPs with their positions that were hits for the MDD-CVD GWAS, but not for any of the specific trait GWASs that made up the latent factor. The x-axis represents the log-transformed p-value in each of the respective GWASs. The dashed line indicates the genome-wide significance threshold of p<5e-8.

The GWAS on the latent MDD-CVD factor resulted in 205 independent genome-wide significant loci (Fig. 3B, independent at R^2^<0.1 and distance≥250kb). We did not observe genomic inflation, indicating that results were not strongly affected by population stratification (LDSC intercept=1.02). Almost three quarters (74.6%) of the significant SNPs showed a high Q heterogeneity, suggesting that their effects were more in line with an independent pathway than a common pathway model. Most of this heterogeneity was due to MDD, as the GWAS for latent CVD without MDD showed fewer SNPs with a high heterogeneity (30.6%; Extended Data Fig. 3, Table S5). For the latent MDD-CVD factor, we filtered out variants that showed significant heterogeneity and considered only variants where the latent MDD-CVD factor was the best model for the follow-up analyses. We retained 72 independent loci underlying the shared genetic liability (Fig. 3B, Table S6). The top SNP after filtering was rs11670056 in the *ELL* gene on chromosome 19, which is part of the transcription elongation factor complex and has previously been associated with a range of CVDs, blood traits, BMI, and educational attainment (enrichment in associations with other traits for significant SNPs are shown in Extended Data Fig. 4). There were 19 top SNPs that were significant eQTLs for one or multiple genes (top 10 are annotated in Fig. 3B, full results are shown in Table S8). Besides *ELL,* multiple genes on chromosome 10 around *INA* and *CNNM2* were identified. *INA* is involved in structural neuron regulation whereas *CNNM2* is involved in ion transportation and has previously been associated with psychiatric as well as cardiovascular traits. From the latent MDD-CVD GWAS summary statistics we extracted seven novel loci that are not among the risk variants in the separate MDD and CVD GWASs (Extended Data Fig. 5, Table S7). The top SNPs in these loci have not been identified in any GWAS before, but four of them have shown suggestive associations (*p*<.05) with metabolic traits (rs11065577, rs11606884, rs2838351, and rs500571).

Using partitioned LDSC, we observed that the heritability of the latent MDD-CVD factor was enriched in genes with expression specific to endothelial and blood vessel tissues, which was also observed for latent CVD, but not for MDD (Fig. 3C, Table S9). To gain deeper insights into brain-specific mechanisms, we leveraged high-resolution human brain single-nucleus RNAseq data^25^ and identified four human brain cell-types that exhibited enriched MDD-CVD heritability, including deep layer corticothalamic and 6b cells, midbrain-derived inhibitory neurons, miscellaneous neurons, and vascular cells (Fig. 3D, Table S10). Notably, three of the four cell-types were uniquely associated with the latent MDD-CVD factor, displaying no enrichment for either latent CVD without MDD or MDD only, suggesting that the genetic variance for MDD-CVD comorbidity has a distinct functional signature.

**Extended Data Figure 6.**
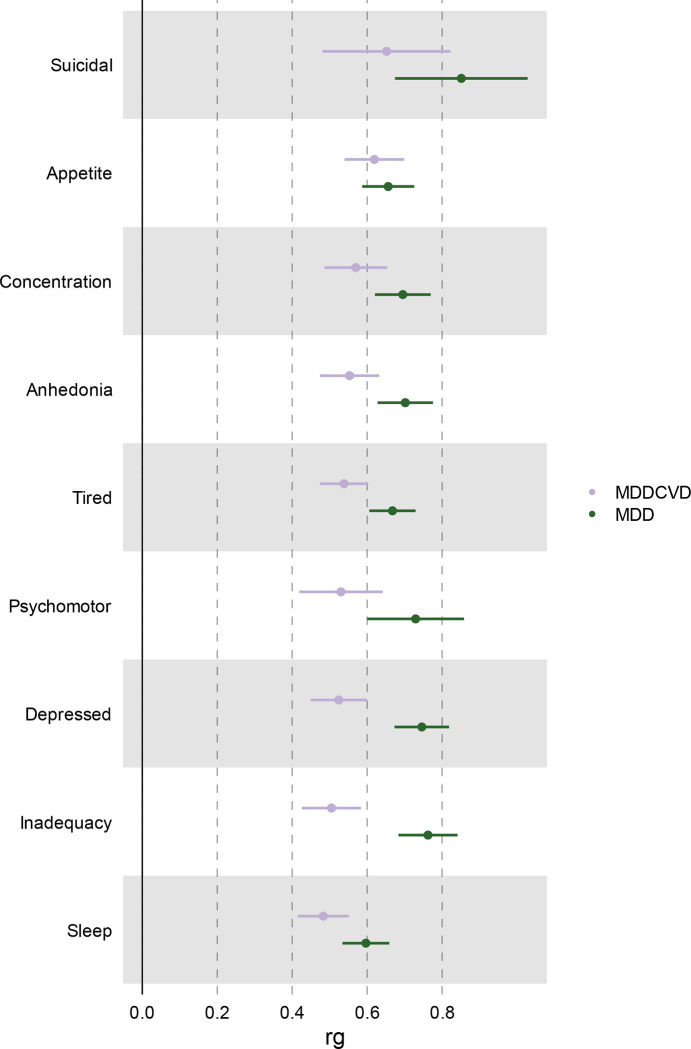
Genetic correlation between MDD/MDD-CVD and the MDD symptoms. MDD-CVD was captured by the GWAS on the latent factor comprising MDD and the CVDs as specified in Genomic SEM. Bars indicate 95% confidence intervals. Genetic correlation results are based on LD Score Regression analysis.

To externally validate the MDD-CVD phenotype, we computed polygenic risk scores (PRS) based on the summary statistics for the latent MDD-CVD factor, as well as for MDD and latent CVD, and found them to significantly predict CVD and MDD diagnoses in UK Biobank (all *p*<2E-13; Table S11; note that source and target samples were overlapping and the R^2^ values should only be interpreted relative to one another). The PRS for latent CVD and the latent MDD-CVD factor explained similar amounts of variance in CVD (Fig. 3E). This is in line with the MiXeR findings (Fig. 2B), suggesting that most causal variants for CVD are shared with MDD. In contrast, as most causal variants for MDD are not shared with CVD, the PRS for the latent MDD-CVD factor explained less than half as much variance in MDD as the MDD PRS. Finally, we assessed whether the latent MDD-CVD factor was differentially associated with specific MDD symptoms, compared to MDD only, and found that most symptoms showed similar correlations with the latent MDD-CVD factor as with MDD only (Extended Data Fig. 6). The largest difference in point estimates was observed for depressed mood and feelings of inadequacy, suggesting that the genetic overlap between MDD and CVD might be driven mainly by the other symptoms.

### Genetic overlap between MDD and risk factors

Next, we aimed to identify risk factors that contribute to the genetic and phenotypic association between MDD and CVD. First, we assessed the genome-wide genetic correlations between MDD and risk factors. Confirming previous findings, we observed strong to moderate genetic correlations of MDD with psychosocial factors, such as loneliness (*r*_*g*_=0.68, SE=0.02), childhood maltreatment (*r*_*g*_=0.55, SE=0.02), and exercise (*r*_*g*_=−0.33, SE=0.02) (Extended Data Fig. 1). Among metabolic risk factors, MDD showed the strongest genetic correlation with type II diabetes (*r*_*g*_=0.19, SE=0.018), followed by levels of high-density lipoprotein cholesterol (*r*_*g*_=−0.14, SE=0.01) and triglycerides (*r*_*g*_=0.18, SE=0.02). MDD showed weak but statistically significant genetic correlations with other metabolic risk factors (i.e., BMI, non-high-density lipoprotein, low-density lipoprotein, and total cholesterol) (*r*_*g*_<0.15). We observed significant genetic correlations for MDD with the inflammatory markers IL-6 (*r*_*g*_=0.22, SE=0.06) and C-reactive protein (*r*_*g*_=0.15, SE=0.02). We did not observe genetic correlation of MDD with blood pressure traits. As a comparison, for CVDs, the largest genetic correlations were found between heart failure and BMI (*r*_*g*_=0.55, SE=0.03) and type II diabetes (*r*_*g*_=0.49, SE=0.03) (Fig. S3, Table S1). Results for MDD symptoms largely followed the pattern of MDD diagnosis, although changed appetite showed stronger genetic correlations with metabolic factors than did MDD diagnosis (Fig. S4, Table S2).

**Extended Data Figure 7.**
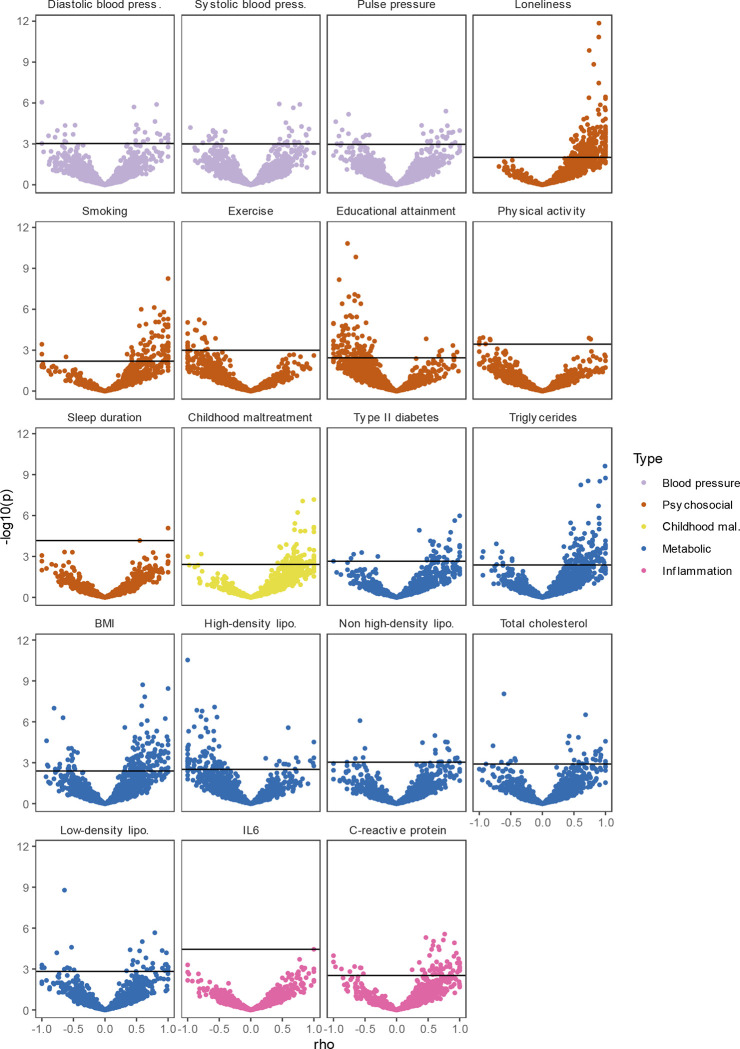
Volcano plots based on LAVA results and shows the genomic loci (dots, N=2,500) with the local genetic correlation between MDD and each of the risk factors (x-axis) and the corresponding *p*-value (y-axis). Loci exceeding the horizontal line are significant at *p*_FDR_<.05.

**Figure 4.**
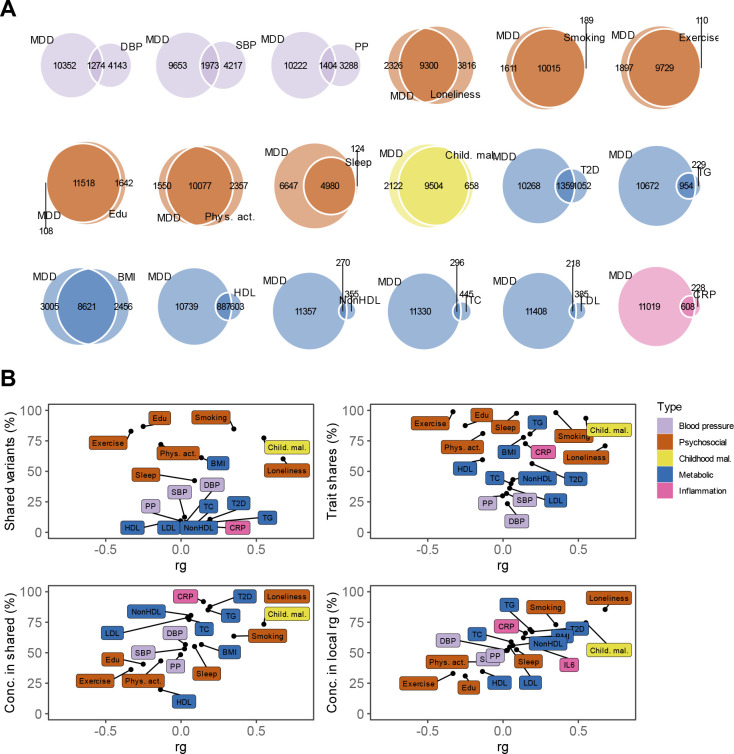
Local and causal-variant level genetic correlations between MDD and risk factors. The legend is shared among all panels. **A** Venn diagrams based on MiXeR results and shows the number of causal variants that are unique to MDD (left circle), the CVD risk factor (non-overlapping part of right circle) or shared between MDD and the CVD risk factor (overlapping part of circles). Note that IL6 was not included because MiXeR did not converge for this trait. **B** Genome-wide genetic correlation estimated by LDSC (rg, x-axis) against the percentage of MDD causal variants that are shared with the CVD risk factor as estimated by MiXeR (top left), the percentage of CVD risk factor causal variants that are shared with MDD (top right), the percentage of CVD risk factor causal variants that are shared with MDD that have the same effect direction (bottom left). The bottom right plot shows the percentage of local genetic correlations from LAVA that are in the same effect direction on the y-axis. Note that for the first 3 plots IL6 was not included because MiXeR did not converge for that trait. DBP=Diastolic Blood Pressure; SBP=Systolic Blood Pressure; PP=Pulse Pressure; Edu=Educational attainment; Phys. Act.=Physical activity; Child. Mal.=Childhood Maltreatment; T2D=Type II Diabetes; TG=Triglycerides; HDL=High-Density Lipoprotein; NonHDL=Non-High-Density Lipoprotein; TC=Total Cholesterol; LDL=Low-Density Lipoprotein; IL6=Interleukin-6; CRP=C-Reactive Protein

Causal variant and local genetic correlation analysis revealed several distinct patterns of polygenic overlap between MDD and risk factors. Psychosocial factors, childhood maltreatment, and BMI showed similar levels of polygenicity to MDD. In addition, they exhibited a large degree of shared risk variants and many local genetic correlations with MDD (Fig. 4A, Extended Data Fig. 7, Table S4–S5). Out of these factors, loneliness and childhood maltreatment also showed high levels of effect direction concordance (93% and 73% of shared causal variants and 85% and 75% of significant local genetic correlations for loneliness and childhood maltreatment were in the same direction; Fig. 4B, correlation coefficients are shown in Table S4–S5). Combined with high polygenicity, such concordance translates to large genome-wide genetic correlations. In contrast, educational attainment, smoking, exercise, physical activity, BMI, and sleep duration had similar levels of polygenicity and a large degree of polygenic overlap with MDD, but low effect direction concordance, suggesting that genome-wide genetic correlations underestimate the polygenic overlap with MDD for these traits.

Genetic risk factors for blood pressure traits showed unique patterns of polygenic overlap in that they were polygenic (>5,000 causal risk variants) but did not overlap strongly with genetic risk factors for MDD (<0.30 of risk variants overlapping). Moreover, risk variants that did overlap showed low degree of effect direction concordance (48%–57% of shared variants in the same direction). We observed 97 significant local genetic correlations for the three blood pressure traits, 60% of which were positive. These findings suggest that MDD and blood pressure share risk variants that exhibit both positive and negative correlations, which are cancelled out in the genome-wide estimate.

Type II diabetes, lipid traits, and C-reactive protein showed low polygenicity (<2,500 causal risk variants). Total cholesterol, non-high-density lipoprotein, and low-density lipoprotein did not share the majority of their risk variants with MDD, and shared variants showed low degrees of effect direction concordance. However, like the CVDs, type II diabetes, triglyceride levels, and C-reactive protein levels shared most of their risk variants with MDD, and these variants showed high degrees of concordance (>85% of shared variants in the same direction). High-density lipoprotein shared most of its risk variants with MDD, in consistently opposite directions.

### Risk factors explain part of the genetic association between MDD and CVD

**Extended Data Figure 8.**
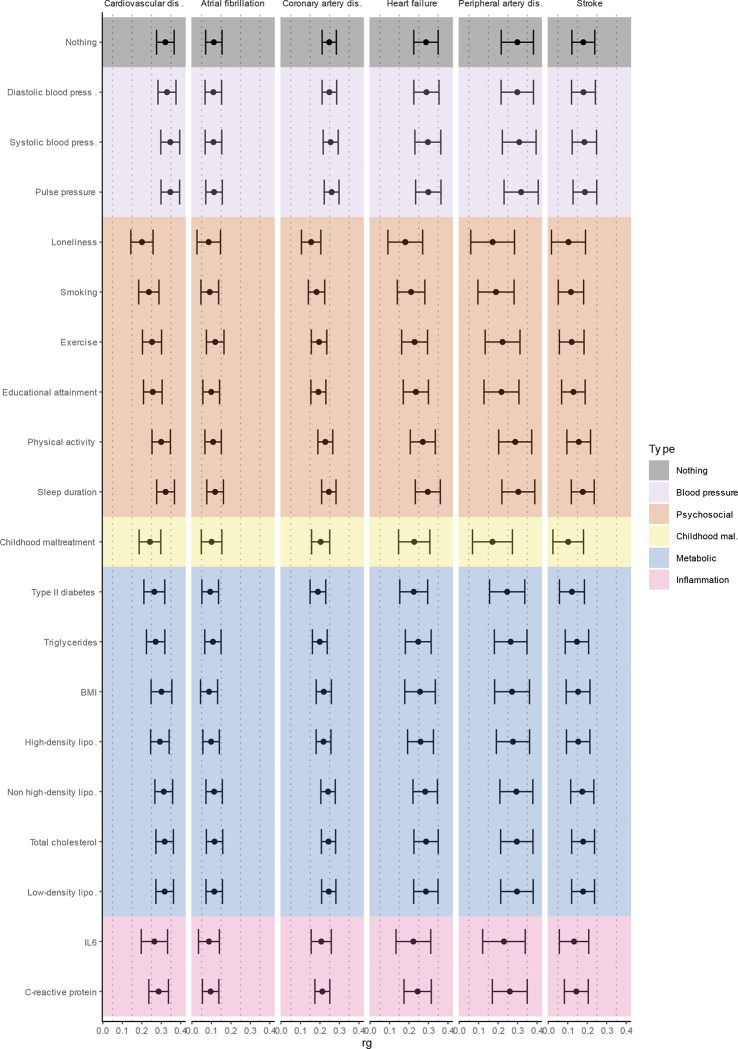
Genetic correlation between MDD and latent CVD (first column) as well as the different CVDs (column 2–6) while adjusting for individual risk factors (rows). Bars represent 95% confidence intervals. The genetic correlation before adjustment is shown in grey (‘nothing’), the other traits are color-coded according to manual categorization. Results from Genomic SEM analyses.

**Figure 5.**
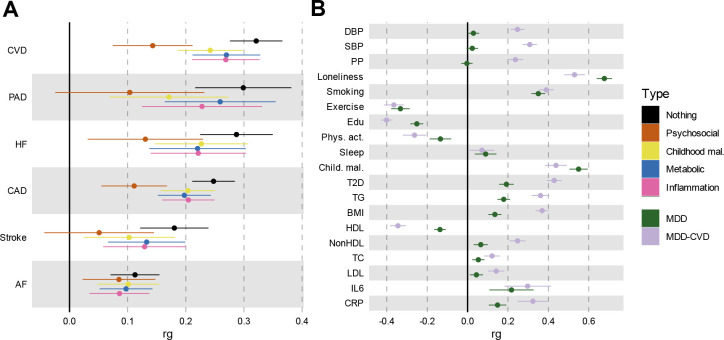
Genome-wide correlations between MDD and risk factors. The legend is shared between panels. **A** genetic correlation between MDD and CVD as before or after adjustment for risk factors (color coded). In black is the estimate without adjustment. **B** Comparison of genetic correlation between MDD (dark green) and the latent CVD-MDD factor (lilac) and individual risk factors. DBP=Diastolic Blood Pressure; SBP=Systolic Blood Pressure; PP=Pulse Pressure; Edu=Educational attainment; Phys. Act.=Physical activity; Child. Mal.=Childhood Maltreatment; T2D=Type II Diabetes; TG=Triglycerides; HDL=High-Density Lipoprotein; NonHDL=Non-High-Density Lipoprotein; TC=Total Cholesterol; LDL=Low-Density Lipoprotein; IL6=Interleukin-6; CRP=C-Reactive Protein

To assess the degree to which risk factors explain the genetic overlap between MDD and CVD, we estimated genetic correlations adjusted for risk factors (individually or as a group) using Genomic SEM (Fig. 5A; results per individual risk factor are shown in Extended Data Fig. 8 and Table S13). The largest reduction in point estimate was observed for the genetic correlation between MDD and peripheral artery disease after adjustment for the group of psychosocial factors, indicating that these risk factors explain much of the genetic association between MDD and peripheral artery disease. Similarly, the genetic correlation between MDD and coronary artery disease, as well as the latent CVD factor, were significantly attenuated after adjusting for psychosocial factors. The reduction was mainly driven by loneliness (Extended Data Fig. 8). Genetic correlations of MDD with peripheral artery disease and stroke were no longer significant after adjusting for psychosocial factors. We also observed some attenuation in the genetic correlation between MDD and CVD after adjusting for childhood maltreatment, metabolic factors, or inflammatory markers, although confidence intervals overlapped.

Next, we specified the risk factors as mediators instead of covariates in the Genomic SEM model and compared the path estimates from MDD to CVD. Observing significant attenuation in the genetic correlation between MDD and CVD when a risk factor is modelled as a mediator rather than a covariate supports the interpretation that the risk factor mediates some of the link between MDD and CVD. For all psychosocial factors together, the inflammation traits, and for type II diabetes, the 95% confidence intervals did not overlap between the mediator and covariate models, suggesting that these risk factors are more likely mediating, rather than confounding, the link between MDD and CVD (Table S14).

Finally, we estimated genetic correlations between the risk factors and the latent MDD-CVD factor (Fig. 5B) and found that the latent MDD-CVD factor was substantially more genetically correlated with blood pressure traits, C-reactive protein levels, and metabolic factors than MDD only, suggesting that these risk factors characterize the genetic liability to MDD-CVD comorbidity rather than to MDD alone.

### Causal pathways linking MDD and CVD

**Figure 6.**
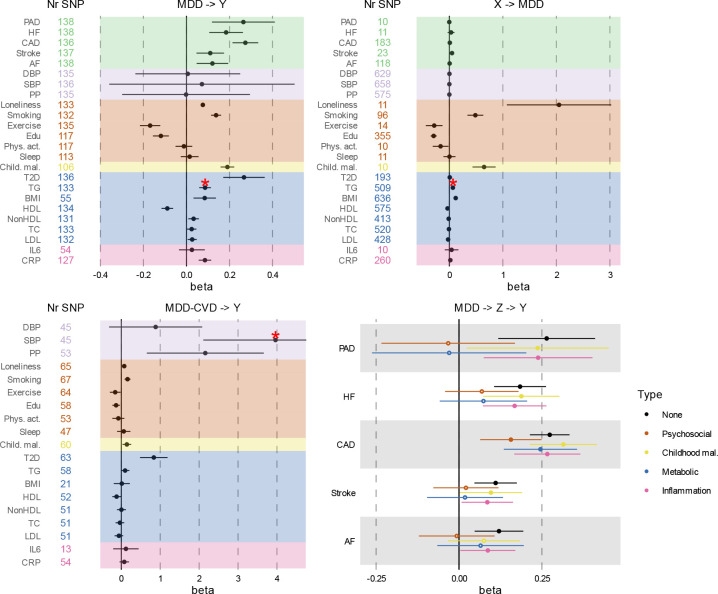
Results from univariable and multivariable MR (IVW estimates). **A** Effect of liability to MDD (exposure) on CVD and risk factors (outcomes). **B** Effects in the opposite direction with MDD as outcome and CVD and risk factors as exposures. **C** Effect of liability to latent MDD-CVD. **D** Attenuation of the effects of MDD on CVDs when adjusting for risk factors. **NOTE:** The x-axis has been scaled in Panel B and C to accommodate visualization of outlier effects. * Indicates significant pleiotropy; causal effect should not be interpreted. PAD=Peripheral Artery Disease; HF=Heart Failure; STR=Stroke; CAD=Coronary Artery Disease; AF=Atrial Fibrillation; DBP=Diastolic Blood Pressure; SBP=Systolic Blood Pressure; PP=Pulse Pressure; Edu=Educational attainment; Phys. Act.=Physical activity; Child. Mal.= Childhood Maltreatment; T2D=Type II Diabetes; TG=Triglycerides; HDL=High-Density Lipoprotein; NonHDL=Non-High-Density Lipoprotein; TC=Total Cholesterol; LDL=Low-Density Lipoprotein; IL6=Interleukin-6; CRP=C-Reactive Protein

We investigated causal relationships between MDD and CVD using two-sample MR. Results from the inverse variance-weighted (IVW) estimator are presented in Fig. 6A–6C (full results Table S15). Instruments were Steiger filtered, i.e., SNPs explaining more variance in the outcome than the exposure were excluded. The results provide support for a causal effect of MDD liability on all CVDs, with the strongest effects observed for coronary and peripheral artery disease. There was no significant pleiotropy for the CVD outcomes, and the results were consistent across weighted median, mode, and Egger sensitivity analyses, providing support for the IVW estimates. Concerning risk factors, we observed no causal effect of MDD liability on blood pressure, physical activity, sleep duration, or IL6. However, increased liability to MDD was associated with increased risk of loneliness, smoking, type II diabetes, and levels of C-reactive protein (Fig. 6A). The results were again consistent across sensitivity analyses. There was significant pleiotropy between MDD and triglycerides, rendering this effect uninterpretable. The average instrument strength of MDD was F=41. Heterogeneity across instruments was significant for most outcomes, suggesting variable effects (Table S15).

We also tested the potential causal effect in the other direction with CVDs and risk factors as exposures and MDD as outcome (Fig. 6B). There was a significant causal effect of liability to stroke, loneliness, smoking, exercise, educational attainment, childhood maltreatment, high-density lipoprotein, and BMI on MDD risk (Fig. 6B), which was robust across the sensitivity analyses. No robust effects were observed for CVDs other than stroke, blood pressure, other metabolic traits, or inflammatory markers.

When using genetic instruments for the latent MDD-CVD factor to predict the risk factors, significant effects were observed for pulse pressure and type II diabetes (Fig. 6C). There was significant pleiotropy for systolic blood pressure. There were small effects on BMI, childhood maltreatment, educational attainment, high-density lipoprotein, exercise, loneliness, and smoking liability, but these were less robust across sensitivity analyses (Table S15). For most outcomes, there was significant instrument heterogeneity.

**Extended Data Figure 9.**
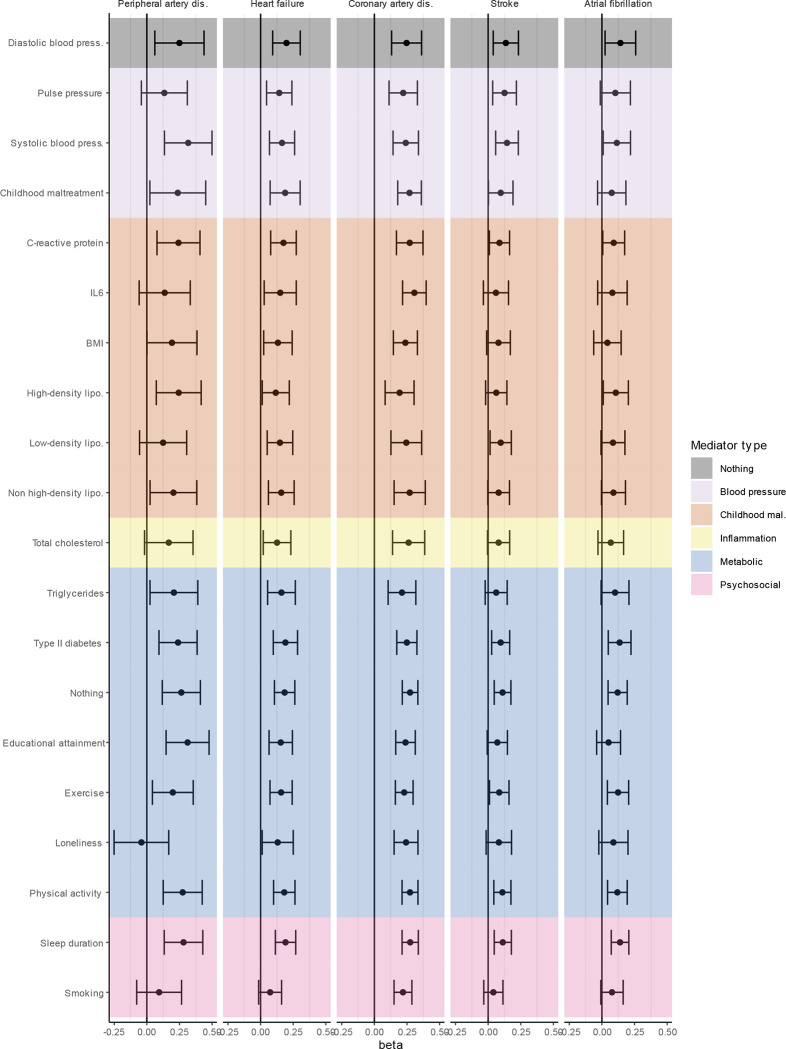
Putatively causal effects of MDD on different CVDs after adjusting for individual risk factors. The estimates are IVW from multivariable MR. Bars represent 95% confidence intervals. The IVW before adjustment is shown in grey (‘nothing’), the other traits are color-coded according to manual categorization.

We extended univariable results with multivariable MR to assess if the causal effects of MDD on CVD could be explained by the risk factors. We included only risk factors that were significantly predicted by MDD in the univariable MR analysis. Fig. 6D shows that, as in the Genomic SEM analyses, the effect of MDD on CVD was attenuated after adjusting for groups of risk factors, although confidence intervals were wide. Results for individual risk factors are shown in Extended Data Fig. 9 and Table S16. The effect of MDD liability on peripheral artery disease, heart failure, stroke, and atrial fibrillation risk was no longer significant after adjusting for the groups of metabolic or psychosocial factors, although the confidence intervals overlapped with those from the unadjusted estimates. Likewise, the effect of MDD on atrial fibrillation was no longer significant after adjusting for childhood maltreatment.

**Extended Data Figure 10.**
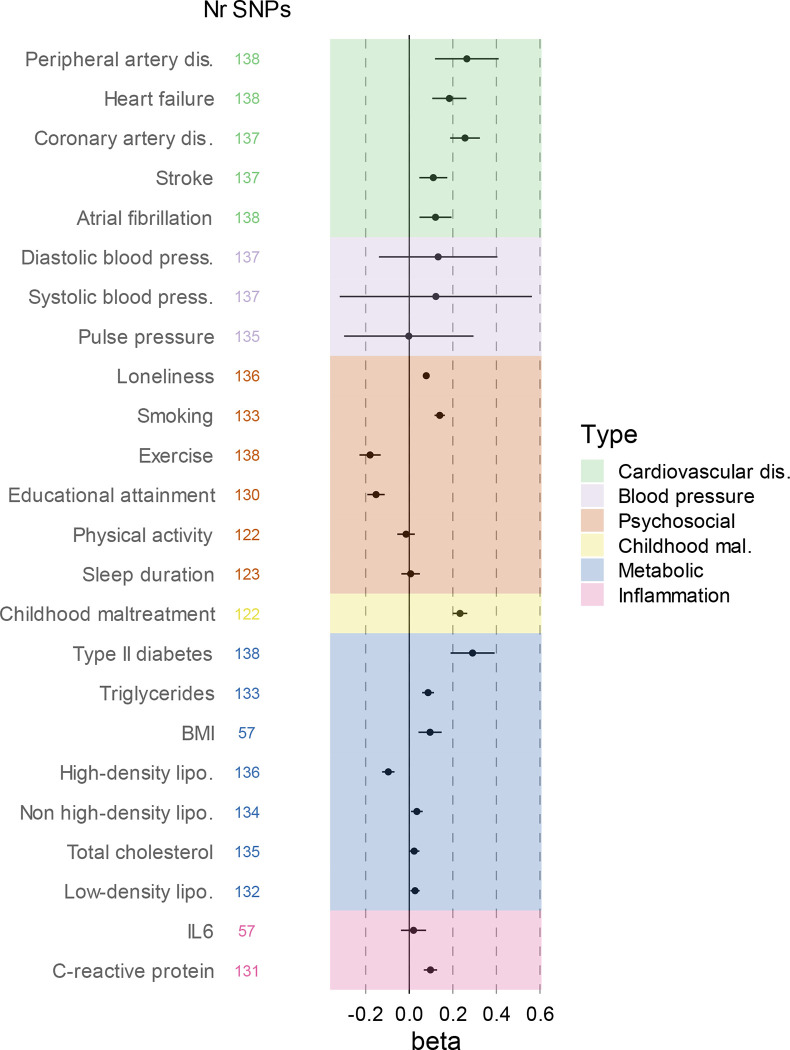
Putatively causal effects of MDD on CVDs and risk factors (univariable) after excluding UK-Biobank. The UK-Biobank sample (responsible for most of the overlap between different GWAS summary statistics) was excluded from the GWAS used to create the MDD instrument. The estimates are IVW from univariable MR after Steiger filtering. Bars represent 95% confidence intervals. Traits are color-coded according to manual categorization. The ‘Nr SNPs’ column gives the number of instrumental SNPs that were present in the outcome GWAS summary statistics.

To control for possible bias due to sample overlap, we repeated the main IVW analyses using MDD summary statistics based on GWAS excluding the UK Biobank sample (the main source of overlap). We observed small differences in the estimates, but the interpretation remained the same for all results (Extended Data Fig. 10, Fig. S5; Table S17). Furthermore, we repeated the analyses using Latent Heritable Confounder (LHC) MR, which is robust to sample overlap^27^. The results pattern was similar, although the point estimates were slightly attenuated for CVD risk (e.g., no longer significant for heart failure and atrial fibrillation) and became stronger for most other traits (Fig. S6). We conclude that although sample overlap impacted the point estimates, the interpretation of results remained similar.

## Discussion

Here, we showed that genetic risk factors for CVD overlap strongly with MDD. We modeled the shared genetic liability between MDD and CVD as a latent factor and showed that, distinct from MDD alone, it is associated with gene expression specific to thalamic and vascular cell-types in the brain and is genetically correlated with immunometabolic factors and blood pressure. Further, we showed that the association between MDD and CVD is partly explained by modifiable risk factors and is likely causal in nature.

In line with previous results^28–31^ we found weak to moderate genome-wide genetic correlations between MDD and CVD. Analysis on the level of overlapping risk variants showed that MDD was substantially more polygenic than the CVD traits and that most risk variants for CVD were in fact shared with MDD and had concordant effect directions. In addition, we found many positive local genetic correlations between MDD and CVD, especially for coronary artery disease. These findings suggest that genetic overlap between MDD and CVD is underestimated in genome-wide correlation analyses.

We modelled the shared liability as a latent MDD-CVD factor and performed a GWAS on the factor. We identified many loci for MDD-CVD, some of which were uniquely associated with the shared liability and not with the constituent traits. We found that heritability for the MDD-CVD latent factor was enriched for genes specifically expressed in vascular braincells, deep layer corticothalamic 6b (projecting to the thalamus), and midbrain-derived inhibitory neurons (predominantly located in the thalamus). This cell-type enrichment signature was not found for MDD or CVD alone, suggesting that a distinct biological mechanism involving thalamic circuits underlies the shared liability to MDD-CVD. Altered thalamic function has indeed been implicated previously in CVD^32–34^ and MDD^35,36^, and white matter integrity in thalamic radiations show associations with aortic area and myocardial wall thickness, suggesting that it has a role in the ‘heart-brain’ connection^37^.

MDD showed a high degree of genetic overlap with risk factors. We showed that MDD was substantially more polygenic than blood pressure traits, lipid traits, and C-reactive protein. Psychosocial traits were equally polygenic and showed high overlap with MDD. Interestingly, the local and variant-level analysis indicated that blood pressure traits shared a substantial proportion of their risk variants with MDD (in line with a previous report^38^), which was masked at the genome-wide level due to their opposing effect directions. Similarly, BMI and lipid traits showed discordant effect directions to MDD in overlapping risk variants as well as opposite directions in local genetic correlations. Finally, C-reactive protein shared almost three quarters of its risk variants with MDD, most of which were in the positive direction, indicating a genetic relationship between C-reactive protein and MDD that was masked by the large polygenicity of MDD. Overall, these findings refine our understanding of the genetic overlap between MDD and risk factors shared between MDD and CVD, and indicate that it is stronger and more complex than has previously been reported.

We estimated genome-wide genetic correlations between MDD and CVD adjusting for risk factors and found that psychosocial factors explain a substantial part of the genetic correlation between MDD and CVD, potentially more as mediators than covariables, and highlight loneliness as an important factor in the relationship between MDD and CVD. Furthermore, we found that, compared to MDD, the latent MDD-CVD factor was characterized by genetic correlations with immunometabolic factors and blood pressure, suggesting that the shared genetic liability to MDD and CVD constitutes an immunometabolic subtype of depression^13,39^.

We found robust support for causal effects of MDD on CVD. Previous two-sample MR studies have observed associations between genetic liability to MDD and risk of coronary artery disease, but results for risk of heart failure have been inconsistent^28,29,40,41^. Another study found an effect of genetic liability to MDD on risk of stroke^42^. Using more recent GWAS data, we confirmed an effect of genetic liability to MDD on coronary artery disease and stroke, and found robust associations for heart failure and peripheral artery disease.

We observed effects of genetic liability to MDD on most of the risk factors. We did not observe associations between genetic liability to MDD and blood pressure traits, although the presence of correlated and anti-correlated genetic components complicates interpretation. Indeed, using genetic instruments for the latent MDD-CVD factor, we did observe strong associations for pulse pressure. Previous MR studies of the association of C-reactive protein and IL-6 levels with MDD risk have also shown inconsistent results^43,44^. We did not find an effect of genetic instruments for inflammatory markers on MDD. However, we did find associations between genetic liability to MDD and increased C-reactive protein levels, lipid levels, and type II diabetes, offering evidence that MDD might lead to long-term dysregulated immunometabolic pathways^39^. Likewise, in line with previous evidence^45^, we observed a causal effect of liability to MDD on smoking, which, in turn, can lead to inflammation.

Analyzing risk factors explaining the causal effect of MDD on CVD, we observed that only the association between genetic liability to MDD and coronary artery disease remained significant after adjusting for psychosocial or metabolic covariates in a multivariable MR analysis. In addition, we found that smoking status attenuates the association between genetic liability to MDD and peripheral artery disease, for which smoking is a particularly strong risk factor^46^. Interestingly, we find that loneliness is an equally important factor explaining the relationship between MDD and peripheral artery disease, as well as heart failure, which is in line with the results from the adjusted genome-wide genetic correlation analysis discussed above.

For most CVDs, no risk factor group could fully explain the genetic association between MDD and the CVD. This suggests that there are other mechanisms at work as well, which are not captured by the genetic data used in the study. For instance, GWAS measure lifetime genetic risk (up to the point of the maximum age in the sample) and cannot capture dynamic processes of cumulative and interactive risk. Relatedly, the statistical genetic tools we employed cannot formally distinguish between mediation and covariation pathways. Future studies should triangulate our findings using longitudinal observational and experimental data. Follow-up studies could also investigate the out-of-sample predictive power (and potential clinical usefulness) of polygenic risk scores for the shared liability to MDD-CVD, as we could not investigate this due to sample overlap in the present study. In addition, to assess generalizability, these findings should be replicated with data from different ancestry sources. The lack of large genetic datasets from non-European populations is a crucial limitation that is widely acknowledged and yet difficult to circumvent. Observational studies have shown that MDD and CVDs could demonstrate different associations depending on ancestry^47,48^, and more genetic and non-genetic research is needed to understand such differences.

To our knowledge, this is the first study moving beyond bivariate genetic overlap to investigate the latent genetic liability shared between MDD and a representative set of CVDs. Our triangulation of genome-wide, local, and variant-level methods provides compelling evidence that MDD and CVD, and their shared risk factors are more strongly overlapping genetically than has previously been reported. Similarly, using genome-wide, variant-level, and genetic instrument methods we show how shared risk factors explain the likely causal association between MDD and CVD. For both MDD and CVDs, we have used updated GWAS summary statistics, with an up to 3-fold increase in sample size compared to previous reports ^28–31^.

This study dissected the architecture of polygenic overlap between MDD and CVD, and their shared risk factors to elucidate mechanisms linking these comorbid diseases. Our findings suggest that the shared genetic liability to MDD and CVD has a distinct biological signature compared to MDD or any CVD separately. Moreover, the shared genetic liability shows stronger genetic correlations with immunometabolic risk factors than MDD alone, in line with the idea of an inflammatory^49^ or immunometabolic^39^ subtype of MDD especially associated with CVDs. We highlight loneliness and smoking as important targets for intervention to reduce the risk of CVD in individuals with MDD. Building on this work, tools can be developed to identify individuals at risk for developing immunometabolic depression and target them for cholesterol-lowering or anti-inflammatory medical interventions.

## Online methods

### Data sources

All data sources were summary statistics from the largest and most recent GWAS to date (see Table 1). Most were publicly available; approval was received for summary statistics containing the Million Veteran Project sample. The MDD symptoms GWAS have not been published, although they have been used in other publications^50^. The symptom GWASs were based on Patient Health Questionnaire (PHQ-9) items measured in UK Biobank and were published online^51^. The disease trait GWASs were mostly based on electronic health record diagnoses. The summary statistics were cleaned using the cleansumstats pipeline (https://github.com/BioPsyk/cleansumstats). SNPs were aligned and harmonized against reference data. Analyses were conducted on the TSD cluster, maintained by the University of Oslo, using singularity containers. Containers are packaged applications with their environmental dependencies that are used to standardize analyses across different sites. Thus, we made sure we used correct software versions and standard parameters^52^.

### Genetic overlap between MDD, CVD, and risk factors

To assess shared liability between MDD, CVD, and risk factors, we estimated genetic overlap on a genome-wide, polygenic, and local level. First, for the genome-wide level, we estimated genetic correlation between each of the traits (Table 1) using LD score regression (LDSC^26^). To assess if there are specific MDD symptoms that could drive these associations, we furthermore estimate genetic correlations between CVD and MDD symptoms as measured with the Patient Health Questionnaire-9 (PHQ-9; Table 1). We excluded the human leukocyte antigen (HLA) region in all analyses. Note that LDSC performs well in the presence of sample overlap.

Second, we used Local Analysis of (co)Variant Association (LAVA^23^) to assess in which regions there were associations between MDD and CVD. The genome was split into 2,500 random, equally sized parts (predefined in the LAVA package) and the genetic correlation between MDD and each of the other traits within each region was estimated.

Third, to investigate polygenic overlap beyond genetic correlation, we use MiXeR v1.3^22^ to assess the number of ‘causal’ variants that are shared between MDD and the other traits. Because it assesses overlap regardless of the effect direction of each variant, it gives a more adequate picture of localized correlations that could cancel each other out when only looking at the genome-wide level. Each analysis was run 20 times, after which the averaged results were plotted in VENN diagrams to visualize the polygenic overlap. We checked the best AIC versus the minimum and the maximum to assess model performance, and did not interpret results when both were more than 1 below zero. To gauge the relationship between polygenic overlap (considering effect direction) and standard genetic correlation (where opposing effects can cancel each other out) we plot the magnitude of the polygenic overlap against the genetic correlation.

### Shared liability to MDD and CVD

To move beyond bivariate association to multivariate overlap, we conducted factor analysis on MDD and the CVDs using Genomic SEM^24^ to assess if there was a single genetic latent factor underlying the associations (‘MDD-CVD’). Genomic SEM uses LDSC to estimate the genetic covariance matrix and uses that in a SEM framework to identify multivariate relationships in the data. A Comparative Fit Index CFI>.90, Standardized Root Mean Square Residual SRMR<.03, and standardized factor loadings *β*>0.3 were set as criteria for acceptable model fit. We defined a CVD factor with the CVDs as indicators as well as a higher-order factor with this CVD factor plus MDD as indicators. The standardized loading of the first indicator (coronary artery disease) was set to 1. The residual variance of the CVD factor was forced to be 0, so that all variance was forced into the MDD -CVD factor. For comparison, we also estimated a common factor model for CVD without MDD, which is visualized in Fig. S2.

Subsequently, we conducted a summary statistics-based GWAS on the MDD-CVD second-order latent factor to identify variants associated with this latent liability. We used the package-default diagonally weighted least squares estimator (DWLS). To derive genome-wide significant independent loci we used the plink clumping procedure as implemented in FUMA^70^, with R=0.6 and distance=250 kb, and reference data from 1000 genomes. To assess the heterogeneity of the SNP effects, we also fit an independent pathway model for each SNP, where each indicator was regressed on the SNP directly instead of forcing the effect through the latent factor. We compare the common pathway χ^2, com^_SNP_ to the independent pathway χ^2, ind^_SNP_ estimates to derive Q_SNP_. For our follow-up analyses, we filtered out all SNPs that had effects that were more consistent with an independent pathway model at Q_pval_<0.05. Using this stringent filter, we focus on SNPs that are shared between MDD and CVD. Using FUMA we overlaid the SNP findings for MDD-CVD to the GWAS-catalog. To define unique loci, we overlaid the independent genomic risk loci (now clumped at R^2^=0.1 and 3000 kb window) for MDD-CVD with the risk loci for the constituent traits (MDD, peripheral artery disease, coronary artery disease, heart failure, stroke, and atrial fibrillation). We used the ‘intersect’ function in bedtools to see if the risk regions were overlapping. If they were independent (according to the clump criteria) they were regarded as novel loci. Also, we identified genes whose regulation is significantly impacted by the top significant SNPs to interpret the biological implications of our findings. For this, we use eQTL estimates from FUMA based on PsychENCODE reference data^71^. Using the same procedures (though without filtering out heterogeneous SNPs) we conducted a GWAS for CVD without MDD.

To externally validate the latent MDD-CVD GWAS results, we computed PRS using LDpred2 (automatic mode with HapMap3 reference data). We compared the MDD-CVD PRS with a PRS based on MDD only, and with a PRS based on latent CVD without MDD. As a target sample we used UK-Biobank. Summary statistics for CVD traits excluding UKB were unavailable, and we chose to leave UKB in for all traits. Sample overlap is likely to lead to overfitting, resulting in an inflation of explained variance. However, this is less of a concern when comparing PRS among themselves, rather than assessing absolute predictive value. As target phenotypes, we extracted CVD and MDD cases according to healthcare registry ICD codes from data field 41270 (see Table S12). We used logistic regression analysis to predict case status from each PRS while controlling for the first 10 principal components for ancestry, sex, and year of birth. Continuous variables were standardized and centered. We estimated Nagelkerke’s R^2^ to capture explained variance in the disease traits.

### Tissue and cell-type analysis

To gain insight into the possible biological mechanisms underlying the common liability to MDD and CVD, we performed a tissue and cell-type analysis using partitioned LDSC. Cell-type identification was based on the top decile of specifically expressed genes (referred to as top decile expression proportion [TDEP] genes). The methodology has been described extensively in previous studies^72–74^. Utilizing snRNAseq data from the Adult Human Brain Atlas^25^, we identified TDEP genes for brain cell-types across 31 superclusters and 461 clusters. Prior to defining specifically expressed genes, we focused on a curated set of 18,090 protein-coding autosomal genes, excluding those in the extended major histocompatibility region (MHC, chr6:25–34mb), and ensuring expression in >1 of the 461 cell-clusters. To establish TDEP genes for 16 canonical human tissues, we utilized bulk RNAseq data from GTEx v8^75^. In line with previous research, we removed tissues with <100 donors and non-natural tissues (e.g., cell lines) as well as testis tissues (expression outlier)^72.^ Prior to analysis in partitioned LDSC, we expanded the boundaries of TDEP genes by 100kb to include possible enhancers or promoters. We then tested the associations between GWAS traits and tissue/cell-types by investigating heritability enrichment within the TDEP genes for each tissue/cell-type. Adjustments were made for 53 baseline LDSC annotations (LDSC v1.0.1) to ensure accuracy and minimize confounding factors.

### Pathways of association between MDD and CVD

#### Mediation in Genomic SEM

We employed several different techniques to assess whether the association between MDD and CVD could be explained by shared risk factors. First, we estimated the genome-wide genetic correlations adjusting for the effects of risk factors using Genomic SEM. To aid interpretation, we added the covariates in groups, controlling for all trait groups separately (psychosocial, childhood maltreatment, metabolic, or inflammation traits; see Table 1). Note that we did not add the blood pressure traits, since they did not show genetic overlap at the genome-wide level. Attenuation of genetic correlation after adjustment was taken to indicate that shared risk factors account for some (or all) of the association between MDD and CVD. Next, we modeled the risk factors explicitly as mediators. In Genomic SEM pathways are modeled directionally, even though statistically no distinction can be made between covariation versus mediation models (i.e., model fit will be identical). If the direct effect is attenuated in the mediation model as compared to the covariate model, we view this as tentative support for the existence of mediation (following procedures reported previously^76^). Finally, we also tested the effects of individual risk factors (both as covariates and mediators) instead of grouping them.

#### Univariable and multivariable Mendelian randomization

To test if the associations between MDD and CVD and risk factors were causal, we used Mendelian Randomization (MR). We assessed the effects of MDD on the CVDs and risk factors, the effect of the CVDs and risk factors on MDD, and the effect of latent MDD-CVD on risk factors. MR uses genetic variants as instrumental variables to test causal effects of an ‘exposure’ on an ‘outcome’. Core assumptions include that the instrumental variables are robustly associated with exposure and are not associated with the outcome (other than through the effect from exposure) or unmeasured confounders. We used the inverse variance weighted (IVW) value in the two-sample MR R-package^77^ as our main estimate. As instrument SNPs, we selected independent GWAS hits at *p<*5e-8, R^2^< 0.001, and distance <5Mb. In the analysis of the effect of genetic instruments of peripheral artery disease, physical activity, childhood maltreatment, and IL6 on MDD risk, we allowed instruments with higher p-values to be able to reach a total of 10 instruments (*p<*1e-5). For the MDD-CVD exposure, we used SNPs that showed no significantly heterogenous effects in the Genomic SEM model (Q_pval_>.05, see above), in order to limit the possibility of pleiotropic effects of this, by nature, heterogenous instrument.

We performed several sensitivity analyses to test and adjust for violation of MR assumptions. All analyses were Steiger filtered, meaning that all SNPs that explained more variance in the outcome than the exposure were excluded as instruments^78^. Weighted median and mode regression were used to correct for effect size outliers that could represent pleiotropic effects^79^. MR-Egger regression was used to assess pleiotropy (pleiotropy leads to a significant intercept) and correct for it (unless I^2^ indicated NOME violation of the NO Measurement Error [NOME] assumption, in which case we did not report MR-Egger results ^80,81^). Second, to assess the strength of our instruments, we used Cochran’s Q to assess heterogeneity in the SNP effects^82^ and the F-statistic to control for weak instrument bias^83^. Third, we performed sensitivity analyses to gauge the effect of sample overlap in the GWASs that were used. Although sample overlap has been suggested to not greatly impact MR results when the source GWASs have a large sample size and the overlap is limited^84^, we wanted to ensure sample overlap did not lead to bias in our findings. We assessed the genetic covariance intercepts for all MDD-trait pairs from the LDSC analyses and observed that most were more than 1 SD away from 0, indicating that sample overlap was present (Table S1). We repeated the analyses with MDD summary statistics leaving out the UK-Biobank sample, which is the sample responsible for most of the overlap and compared the results. Also, we repeated the analyses using Latent Heritable Confounder MR (LHC-MR^27^), which aims to correct for the presence of unmeasured heritable confounders as well as sample overlap.

Subsequently, we test the adjusted pathways using multivariable MR (MVMR) analyses. The difference with the mediation test in Genomic SEM is that we now use instrumental variables that corroborate a causal interpretation. Because MVMR relies on simple regression analysis, it cannot statistically test mediation; instead, the causal estimate is adjusted for the risk factor. To support a directional interpretation, we included only risk factors as mediators that were significantly affected by MDD according to the univariable analysis results. In MVMR, only an IVW estimate can be derived. Steiger filtering was performed on the exposure-outcome association. These analyses were not replicated in LHC, which does not accommodate multivariable analyses. For ease of interpretation, we again grouped the mediators and adjusted for all mediators in a group concurrently. Additionally, we performed analyses adjusting for single mediators. We selected instruments for each variable in a model by a clumping step with the same parameters as in the univariate MR case (*p*<5e-8, R^2^< 0.001). We then combined instruments for all variables in a model into a single set of instruments and performed another clumping step with the same parameters. These instruments were then aligned to the same effect allele. We estimated the effect of genetic liability to MDD on CVD traits adjusting for covariates using multivariate MR with the MVMR R-package^85^.

## Extended Data

## Supplementary Material

Supplement 1

Supplement 2

Supplement 3

Supplement 4

Supplement 5

Supplement 6

Supplement 7

Supplement 8

Supplement 9

Supplement 10

Supplement 11

Supplement 12

Supplement 13

## Figures and Tables

**Figure 1.**
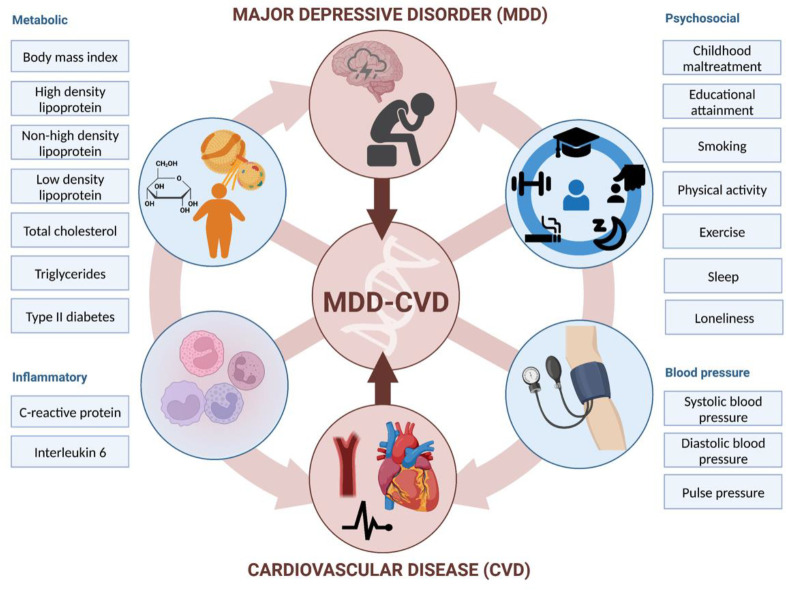
Graphical abstract illustrating the study approach. The comorbidity between MDD and CVD is investigated using genetic and causal inference methods, including assessing overlap with and mediation through shared risk factors (metabolic, psychosocial, inflammatory, and blood pressure). Created with BioRender.com.

**Table 1.** Traits with their source GWAS. The ‘Ref’ column includes the reference as numbered in the bibliography. For binary traits, sample prevalence (prev) is given. The SNP-based heritability (h^2^_SNP_) is given as provided in the source article*.

Trait group	Ref	Phenotype	Name in plots	Contains UKB	N	Sample prev	h^2^_SNP_	Consortia	Year	doi
CVD	53	Atrial fibrillation	Atrial fibrillation	yes	1,030,836	0.06	0.42	AFGen, Broad AF	2018	10.1038/s41588-018-0133-9
CVD	54	Coronary artery disease	Coronary artery disease	yes	1,165,720	0.16	0.16	CARDIoGRA MplusC4D	2022	10.1038/s41588-022-01233-6
CVD	55	Heart Failure	Heart Failure	yes	977,323	0.05	0.09	HERMES	2020	10.1038/s41467-019-13690-5
CVD	56	Stroke	Stroke	yes	1,308,460	0.05	0.01	GIGASTROKE	2022	10.1038/s41586-022-05165-3
CVD	57	Peripheral artery disease	Peripheral artery disease	yes	449,548	0.03	0.55	MVP/UKB	2021	10.1161/CIRCGEN.119.002862
MDD	4	Major depressive disorder	MDD	yes	1,035,760	0.28	0.07	iPSCYH/ PGC	2022	10.1038/s41591-023-02352-1
MDD symptoms	50	Anhedonia	Anhedonia	yes	117,757	NA	0.05*	(UKB)	NA	Neale Lab UK Biobank. https://www.nealelab.is/uk-biobank/
MDD symptoms	50	Depressed mood	Depressed	yes	117,656	NA	0.04*	(UKB)	NA	Neale Lab UK Biobank. https://www.nealelab.is/uk-biobank/
MDD symptoms	50	Sleep	Sleep	yes	117,822	NA	0.06*	(UKB)	NA	Neale Lab UK Biobank. https://www.nealelab.is/uk-biobank/
MDD symptoms	50	Tiredness	Tired	yes	117,828	NA	0.07*	(UKB)	NA	Neale Lab UK Biobank. https://www.nealelab.is/uk-biobank/
MDD symptoms	50	Changes in appetite	Appetite	yes	117,907	NA	0.05*	(UKB)	NA	Neale Lab UK Biobank. https://www.nealelab.is/uk-biobank/
MDD symptoms	50	Feelings of inadequacy	Inadequacy	yes	117,502	NA	0.03*	(UKB)	NA	Neale Lab UK Biobank. https://www.nealelab.is/uk-biobank/
MDD symptoms	50	Concentration problems	Concentration	yes	117,899	NA	0.05*	(UKB)	NA	Neale Lab UK Biobank. https://www.nealelab.is/uk-biobank/
MDD symptoms	50	Psychomotor changes	Psychomotor	yes	117,868	NA	0.02*	(UKB)	NA	Neale Lab UK Biobank. https://www.nealelab.is/uk-biobank/
MDD symptoms	50	Suicidality	Suicidal	yes	117,177	NA	0.02*	(UKB)	NA	Neale Lab UK Biobank. https://www.nealelab.is/uk-biobank/
Childhood maltreatment	58	Childhood Maltreatment	Childhood maltreatment	yes	150,290	NA	0.06	PGC	2020	10.1038/s41398-020-0706-0
Risk factors: psychosocial	59	Educational attainment	Educational attainment	yes	766,345	NA	0.12	SSGAC	2018	10.1038/s41588-018-0147-3
Risk factors: psychosocial	60	Ever regular smoker	Smoking	no	848,460	0.46	0.08	GSCAN	2019	10.1038/s41588-018-0307-5
Risk factors: psychosocial	61	Physical activity (accelerometer)	Physical activity	yes	91,105	NA	0.21	(UKB)	2018	10.1038/s41467-018-07743-4
Risk factors: psychosocial	62	Self-reported activity	Exercise	yes	703,901	0.47	0.04 0.08	NA	2022	10.1038/s41588-022-01165-1
Risk factors: psychosocial	61	Sleep duration	Sleep duration	yes	91,105	NA	0.19	(UKB)	2018	10.1038/s41467-018-07743-4
Risk factors: psychosocial	63	Loneliness	Loneliness	yes	452,302	NA	0.04	(UKB)	2018	10.1038/s41467-018-04930-1
Risk factors: metabolic	64	Body Mass Index	BMI	yes	795,640	NA	0.22	GIANT	2018	10.1093/hmg/ddy271
Risk factors: metabolic	65	High-density lipoprotein	High-density lipo.	yes	1,244,580	NA	0.11*	GLGC	2021	10.1038/s41586-021-04064-3
Risk factors: metabolic	65	Non-high-density lipoprotein	Non-high-density lipo.	yes	926,571	NA	0.08*	GLGC	2021	10.1038/s41586-021-04064-3
Risk factors: metabolic	65	Low-density lipoprotein	Low-density lipo.	yes	1,231,289	NA	0.07*	GLGC	2021	10.1038/s41586-021-04064-3
Risk factors: metabolic	65	Total cholesterol	Total cholesterol	yes	1,253,277	NA	0.08*	GLGC	2021	10.1038/s41586-021-04064-3
Risk factors: metabolic	65	Triglycerides	Triglycerides	yes	1,320,016	NA	0.10*	GLGC	2021	10.1038/s41586-021-04064-3
Risk factors: metabolic	66	Type II diabetes	Type II diabetes	yes	80,154	853,816	0.05*	DIAMANTE	2022	10.1038/s41588-022-01058-3
Risk factors: inflammation	67	Interleukin 6	IL6	no	52,654	NA	0.04	CHARGE	2021	10.1093/hmg/ddab023
Risk factors: inflammation	68	C-reactive protein	C-reactive protein	yes	575,531	NA	0.13	CHARGE	2022	10.1038/s41467-022-29650-5
Risk factors: blood pressure	69	Diastolic Blood Pressure	Diastolic Blood Press.	yes	757,601	NA	0.21	UKB, ICBP	2018	10.1038/s41588-018-0205-x
Risk factors: blood pressure	69	Pulse Pressure	Pulse Pressure	yes	745,820	NA	0.19	UKB, ICBP	2018	10.1038/s41588-018-0205-x
Risk factors: blood pressure	69	Systolic Blood Pressure	Systolic Blood Press.	yes	745,820	NA	0.21	UKB, ICBP	2018	10.1038/s41588-018-0205-x

* When it was not reported in the source article, we include the observed scale h^2^sNP estimated by LDSC for the genetic correlation analyses. Note that different studies have used different methods to derive h^2^sNP.

*UKB=UK Biobank; sample prev=sample prevalence; CVD=cardiovascular disease;* CARDIoGRAMplusC4D=*(Coronary ARtery Disease Genome wide Replication and Meta-analysis (CARDIoGRAM) plus The Coronary Artery Disease (C4D) Genetics); HERMES=Heart failure Molecular Epidemiology for Therapeutic targets; MVP=the Million Veteran Program; MDD=major depressive disorder; PGC=Psychiatric Genomics Consortium; SSGAC=Social Science Genetic Association Consortium; GSCAN=GWAS & Sequencing Consortium of Alcohol and Nicotine use; GiANT=The Genetic investigation of Anthropometric Traits consortium; GLGC=Global Lipids Genetics Consortium; DiAMANTE=Diabetes Meta-Analysis of Trans-Ethnic association studies; inflamm=inflammation; CHARGE=Cohorts for Heart and Aging Research in Genomic Epidemiology; blood pres=blood pressure; iCBP=international Consortium for Blood Pressure.*
