# Supplementary material for: Distinct biological signature and modifiable risk factors underly the comorbidity between major depressive disorder and cardiovascular disease
Source: medRxiv. 2023 Sep 1:2023.09.01.23294931. Preprint. [Version 1] doi: 10.1101/2023.09.01.23294931 (PMC10491387; doi:10.1101/2023.09.01.23294931)
Supplement: Supplementary file 1 [file media-1.pdf]

Fig S1. Genetic correlation matrix for MDD, CVDs, and risk factors.

Cell colour indicates the strength of the genetic correlation. Corresponding statistics are in Table S1.

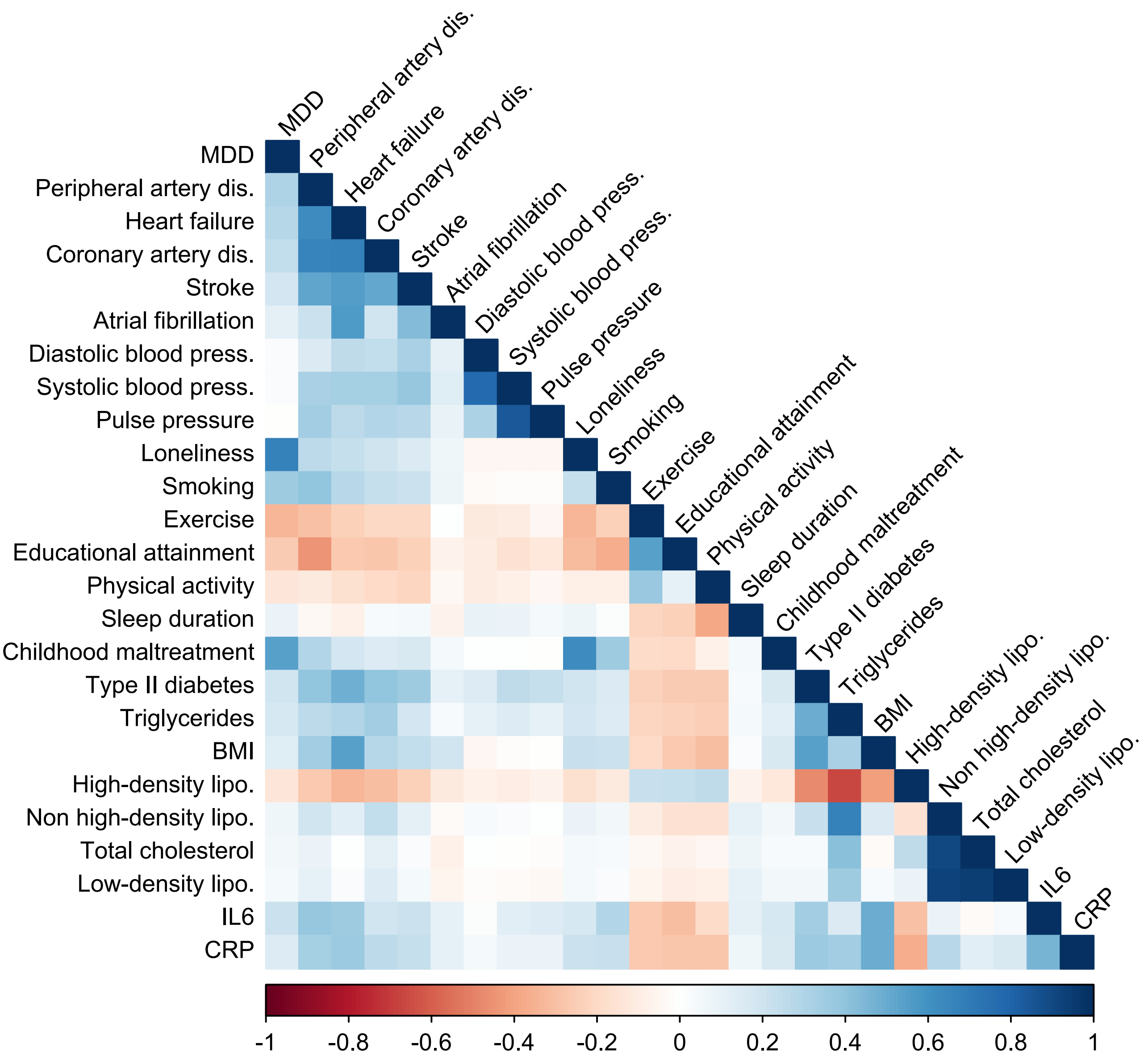

Fig S2. Structural equation model for a common latent CVD factor.

Latent CVD model, defined by heart failure (HF), peripheral artery disease (PAD), stroke (STR), and coronary artery disease (CAD). All ‘observed’ traits are based on GWAS summary statistics. Results are from confirmatory factor analysis in Genomic SEM, and standardized factor loadings are given for each path. Circular dashed arrows give the trait variance.

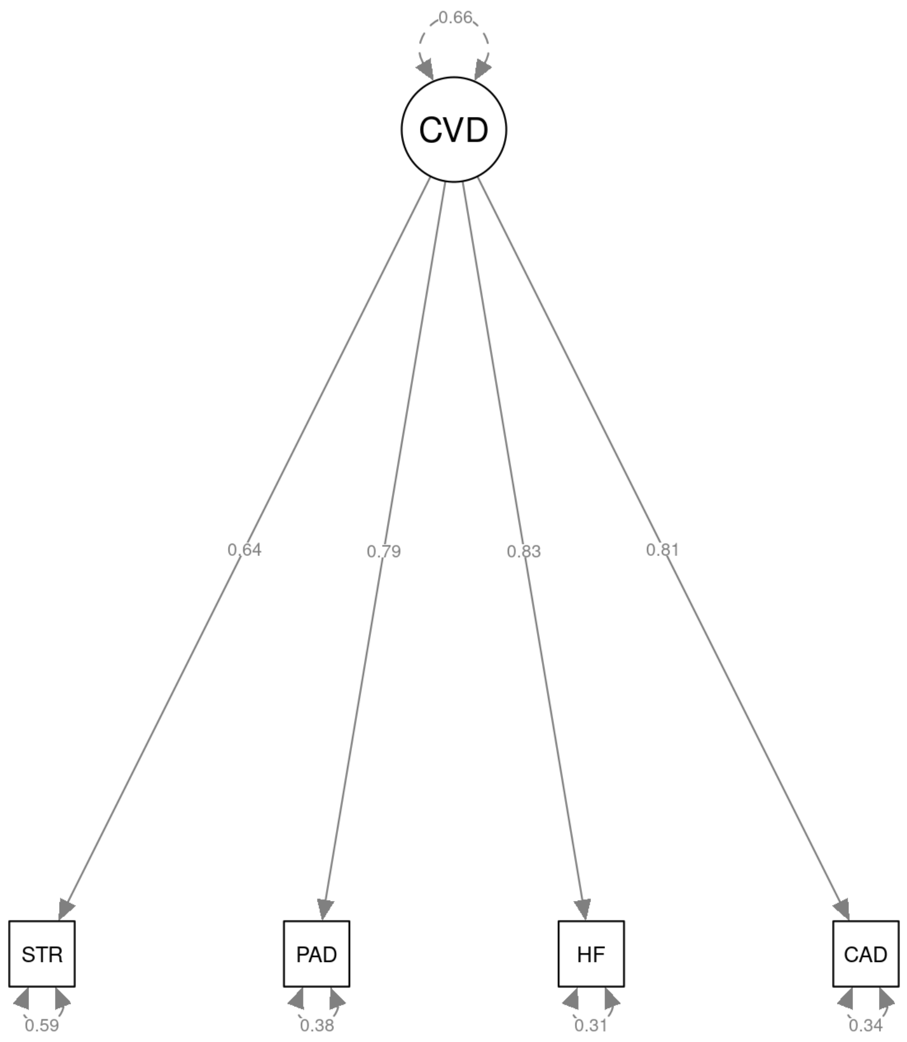

Fig S3. Genetic correlation between different CVDs and risk factors.

Bars represent 95% confidence intervals. Point estimates without fill color are not significantly different from zero.

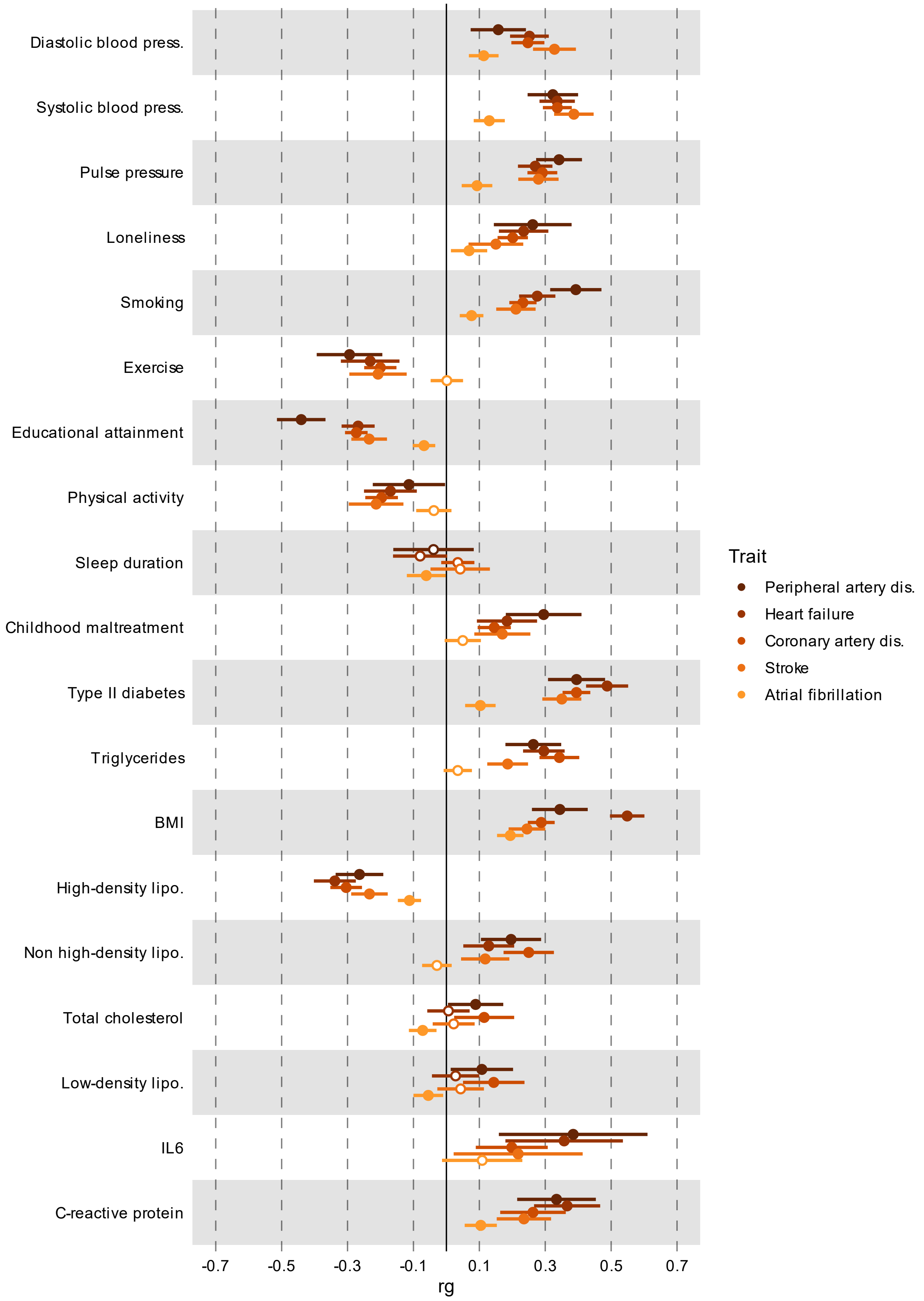

Fig S4. Genetic correlation between the risk factors and MDD symptoms.

Bars represent 95% confidence intervals.

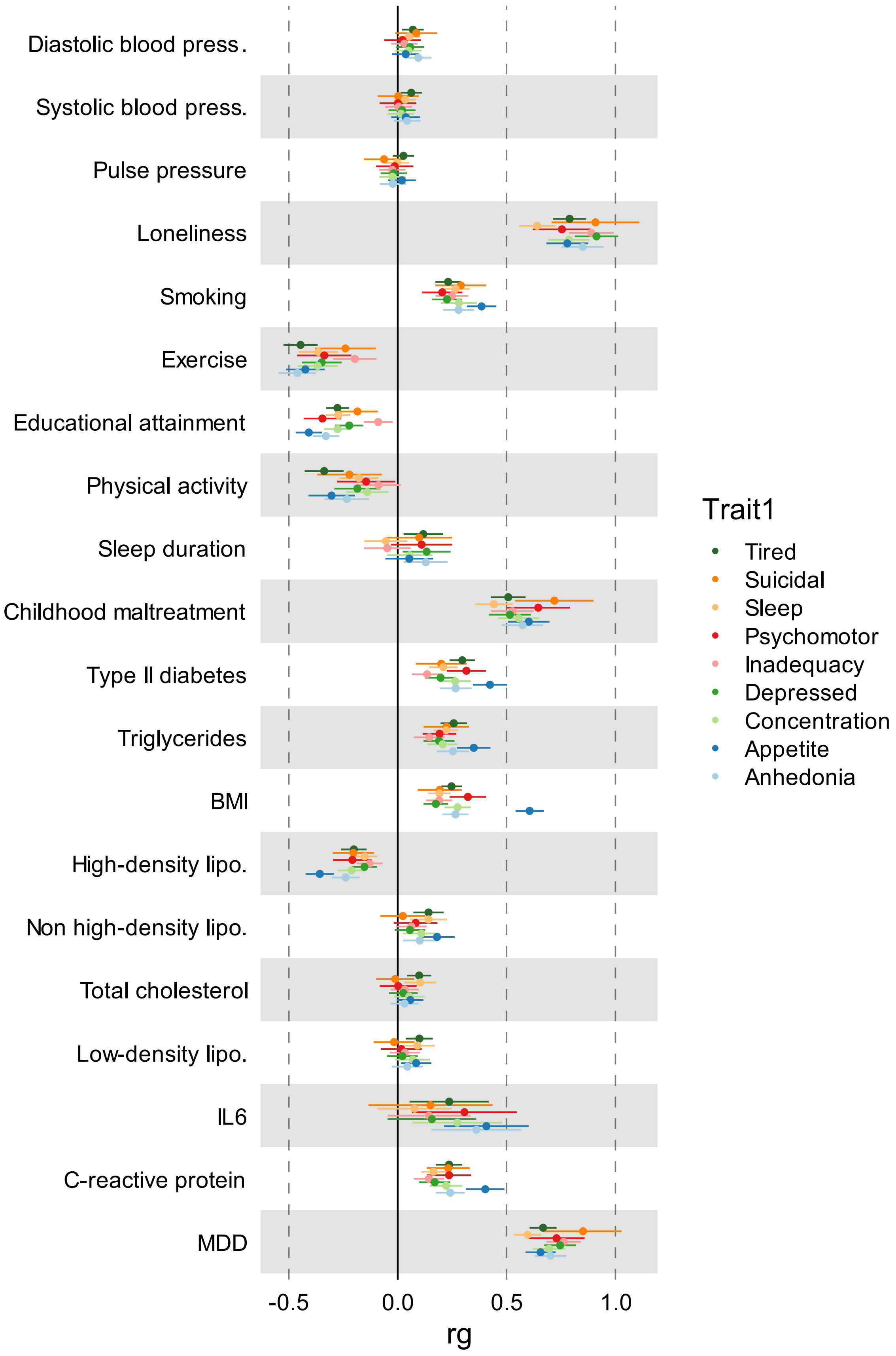

Fig S5. Mendelian randomization effects of CVDs and risk factors on MDD (univariable) after excluding UK-Biobank.

The UK-Biobank sample (responsible for most of the overlap between different GWAS summary statistics) was excluded from the GWAS used to represent the MDD outcome. The estimates are IVW from univariable MR after Steiger filtering. Bars represent 95% confidence intervals. Traits are color-coded according to manual categorization. The ‘Nr SNPs’ column gives the number of instrumental SNPs that were present in the outcome GWAS summary statistics.

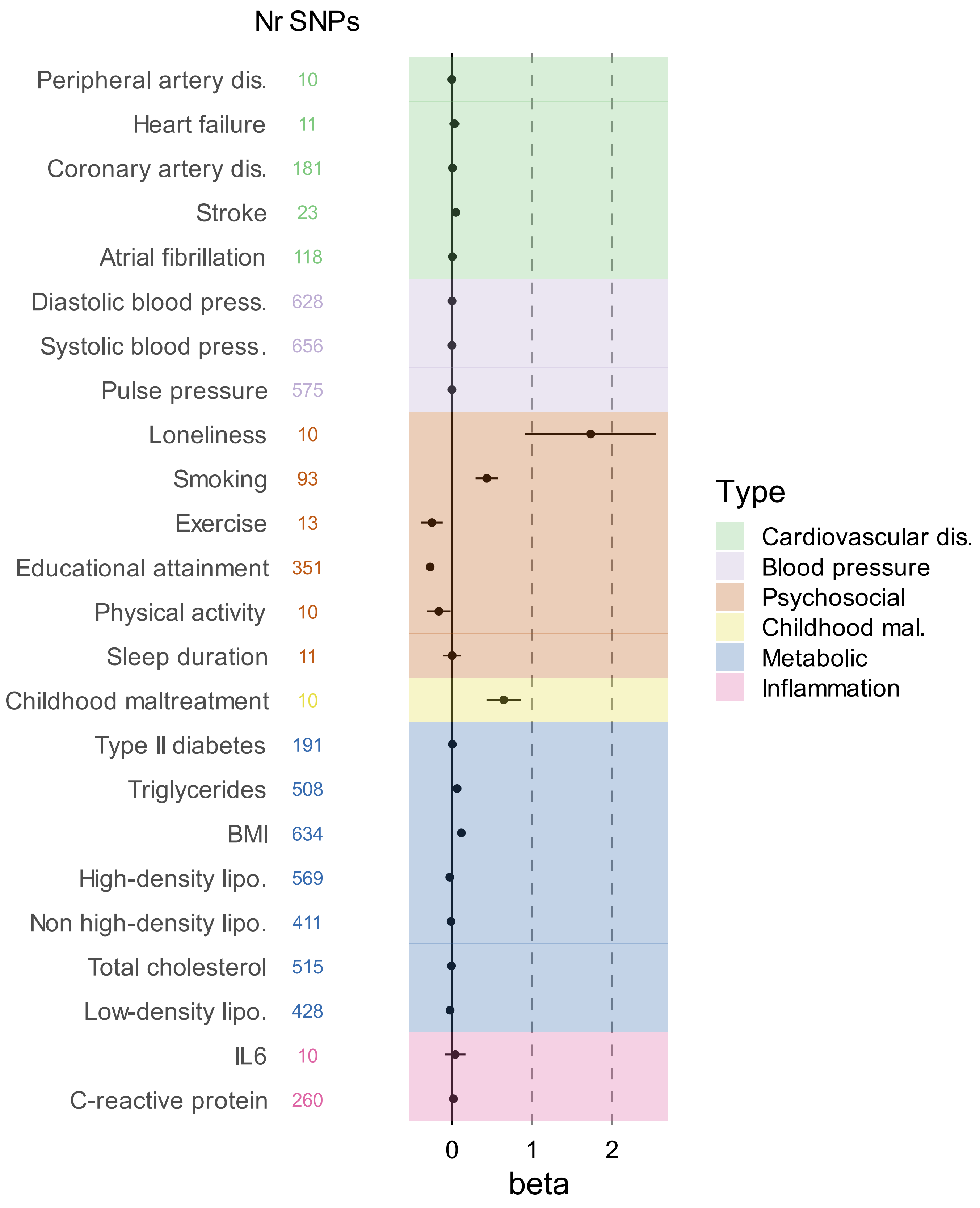

Fig S6. Mendelian randomization effects of MDD on CVDs and risk factors (univariable) according to standard MR versus LHC.

IVW estimate is presented for the standard univariable two-sample MR method after Steiger filtering (in black) as well as for Latent Heritable Confounder MR (LHC; in gray). Bars represent 95% confidence intervals. Traits are color-coded according to manual categorization.

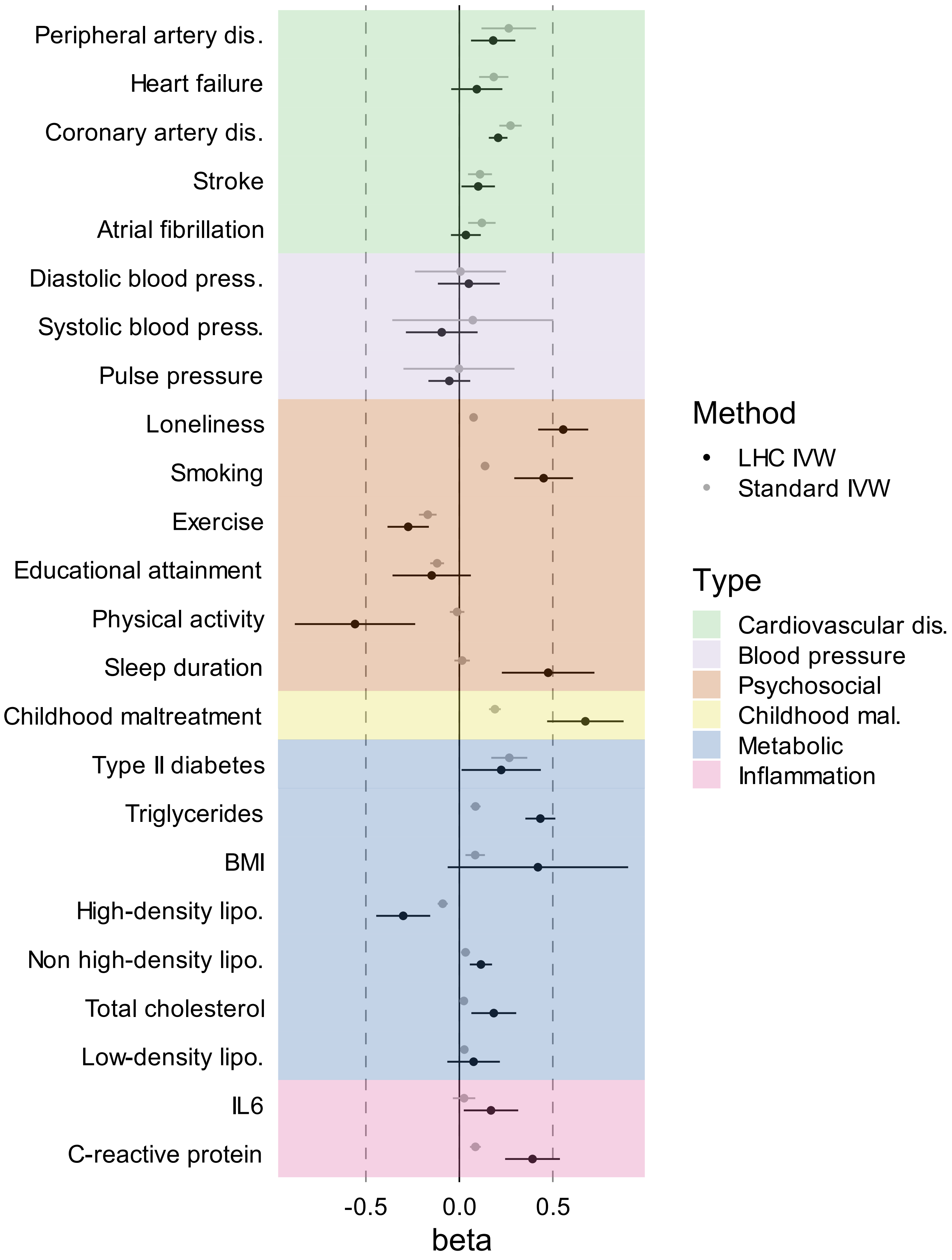

Table S1. Full genetic correlation results from LDscore Regression between all traits, including MDD, CVD, and risk factors.

Excel sheet TableS1

Table S2. Full genetic correlation results from LDscore Regression between MDD symptoms and all other traits (CVD, MDD, and risk factors)

Excel sheet TableS2

Table S3. Results from LAVA showing genomic partitions with a significant (after correction) local genetic correlation (Rho). The number of SNPs per locus is given, with the number of principal components capturing the vairance at that locus. R2 is a measure of variance explained in the phenotype by signal at the locus. For Rho and R2 bounds for the 95% confidence intervals are given. The p and FDR-corrected p-value corresponding to Rho for the association within each partition is given.

Excel sheet TableS3

Table S4. Results from MiXeR showing overlap between the causal variants for MDD and causal variants for CVD/ risk factors (Trait). Rho is the genetic correlation between the causal variants for MDD and the causal variants for the other trait. Rg is the corresponding genome-wide genetic correlation. % Concordance indicates how much of the causal variants that are shared between the traits have effects in the same direction. N unique indicates the number of non-shared SNPs and N overlap is the number of shared SNPs. The overlap is expressed as a percentage of all causal variants for the trait (% trait shared) as well as a percentage of the number of variants for MDD (%MDD shared).

Excel sheet TableS4

Table S5. Lead SNPs per locus associated with latent CVD (significant at 5E-8, independent at R2<0.1). Loci less than 250 kb apart are merged. Per lead SNP, the nearest gene is given with its distance to the lead SNP. The Combined Annotation Dependent Depletion (CADD) score indicates the deleteriousness of the lead SNP. Other SNPs in this locus are listed in the last two columns.

Excel sheet TableS5

Table S6. Lead SNPs per locus associated with latent CVD-MDD (significant at 5E-8, independent at R2<0.1). Loci less than 250 kb apart are merged. Per lead SNP, the nearest gene is given with its distance to the lead SNP. The Combined Annotation Dependent Depletion (CADD) score indicates the deleteriousness of the lead SNP. Other SNPs in this locus are listed in the last two columns.

Excel sheet TableS6

Table S7. Novel loci for MDD-CVD that were not identified as risk variants for either of the MDD or separate CVD traits. The top SNP was looked up in GWAS central for previous suggestive associations with other traits at p<.05. Minor allele frequency was retrieved for a European ancestry population (TOPMED). Ggenes in the region were looked up using UCSC's Genome Browser.

SNP	CHR	BP start	BP stop	Genes in region (UCSC)	Key associations SNPs (GWAS central, p<.05)	MAF (TOPMED)
rs11065577	chr12	1.22E+08	1.22E+08	KDM2B	BMI, pulmonary, proinsulin, glycemic	0.29
rs11606884	chr11	49437043	50776940	OR4C12, OR4C13	waist-hip ratio, pulmonary function	0.07
rs2838351	chr21	44931965	45132764	HSF2BP, RRP1B	BMI, pulmonary, glycemic, Hodgkin	0.28
rs34536536	chr17	41095586	41458020	BRCA1, NBR1, TMEM106A, RND2, RPL25, VAT1, PTGES3L, RUNDC1, PTGES3L-AARSD1	-	0.17
rs35864506	chr4	1E+08	1E+08	-	-	0.12
rs500571	chr11	82933549	83334530	CCDC90B, ANKRD42, DLG2	glycemic	0.31
rs76224062	chr2	1.86E+08	1.86E+08	-	-	0.19

Table S8. Results from eQTL mapping of the CVD-MDD GWAS. Results are reported for genome-wide significant, independent loci. Reference data are from PsychENCODE (http://resource.psychencode.org). For each SNP, the p-value from the CVD-MDD GWAS is given (p GWAS), along with the gene name for which it was a singificant eQTL (Gene and Gene (Ensembl ID) and its position and risk allele. The eQTL parameter is filtered on >0.1 FPKM in at least 10 samples. The Direction column indicates if the SNP significantly in- or decreased the expression of the gene.

SNP	p GWAS	Gene	Gene (Ensembl ID)	CHR	BP	Risk allele	eQTL parameter	p eQTL (FDR)	Direction
rs11670056	1.90E-14	ELL	ENSG00000105656	19	18589943	T	0.155	0.004	+
rs12414777	6.53E-13	AS3MT	ENSG00000214435	10	104697781	C	-0.255	1.75E-22	+
rs12414777	6.53E-13	C10orf32	ENSG00000166275	10	104697781	C	-0.197	5.40E-22	+
rs12414777	6.53E-13	INA	ENSG00000148798	10	104697781	C	0.081	1.57E-14	-
rs12414777	6.53E-13	CNNM2	ENSG00000148842	10	104697781	C	0.102	3.54E-05	-
rs2100053	2.32E-12	C2orf82	ENSG00000182600	2	233719516	G	0.499	4.52E-125	-
rs13131930	3.54E-12	GUCY1A3	ENSG00000164116	4	156614184	C	0.117	3.84E-17	-
rs58749629	5.70E-11	WFDC3	ENSG00000124116	20	44571317	A	-0.222	9.97E-07	-
rs4678361	1.64E-10	PCCB	ENSG00000114054	3	135833005	G	0.102	5.33E-08	+
rs2764203	4.98E-10	C6orf106	ENSG00000196821	6	34708901	G	0.245	1.20E-35	+
rs2764203	4.98E-10	SNRPC	ENSG00000124562	6	34708901	G	-0.163	3.32E-27	-
rs2764203	4.98E-10	UHRF1BP1	ENSG00000065060	6	34708901	G	0.177	4.00E-18	+
rs431611	1.10E-09	ZNF831	ENSG00000124203	20	57710647	G	0.051	0.017	-
rs2350632	1.14E-09	TOM1L2	ENSG00000175662	17	17573037	C	0.067	9.50E-08	-
rs2350632	1.14E-09	LRRC48	ENSG00000171962	17	17573037	C	-0.063	0.029	+
rs357466	1.26E-09	DHX36	ENSG00000174953	3	153902868	G	-0.142	5.73E-13	-
rs357466	1.26E-09	ARHGEF26	ENSG00000114790	3	153902868	G	-0.135	6.45E-09	-
rs143499091	1.36E-09	NCOA6	ENSG00000198646	20	33465370	C	-0.170	4.42E-08	+
rs143499091	1.36E-09	PROCR	ENSG00000101000	20	33465370	C	-0.167	0.001	+
rs11670143	1.56E-09	ATP5SL	ENSG00000105341	19	41864971	A	0.195	2.28E-33	-
rs11673093	2.21E-09	KLC3	ENSG00000104892	19	45742094	A	0.125	0.010	+
rs4836381	4.89E-09	FBN2	ENSG00000138829	5	127922270	G	0.077	0.033	+
rs2306363	9.46E-09	RNASEH2C	ENSG00000172922	11	65405600	G	0.096	0.004	-
rs2306363	9.46E-09	SNX32	ENSG00000172803	11	65405600	G	-0.061	0.040	+
rs12145098	1.65E-08	GABPB2	ENSG00000143458	1	151655627	A	-0.081	0.036	+
rs34536536	2.38E-08	NBR1	ENSG00000188554	17	41443671	C	-0.093	4.69E-05	-
rs11065577	3.52E-08	ANAPC5	ENSG00000089053	12	121879592	C	0.051	0.005	-
rs35864506	3.62E-08	ADH1B	ENSG00000196616	4	100301700	G	-0.122	0.006	-

Table S9. Results on tissue enrichment for latent CVD-MDD, latent CVD (without MDD), and MDD from partioned LDscore regression.

Excel sheet TableS9

Table S10. Results on brain cell type enrichment for latent CVD-MDD, latent CVD (without MDD), and MDD from partioned LDscore regression. The FDR-corrected p-value (p.fdr) was used to determine significance.

Excel sheet TableS10

Table S11. Results of regression analysis of three different polygenic risk scores (PRS) on MDD and CVD outcomes. Models were adjusted for birth year, sex, and the first 10 principal components for ancestry. Nore that the PRS were based on GWAS that in most cases included the target sample (UK Biobank), so that the effect sizes are probably biased upwards.

PRS	Outcome	Beta	SE	Z	P	% R2
CVD	AF	0.16	0.01	25.06	1E-138	0.36
CVD	CAD	0.70	0.01	120.25	<5E-324	7.36
CVD	HF	0.43	0.01	47.57	<5E-324	2.14
CVD	MDD	0.09	0.01	12.66	9E-37	0.11
CVD	PAD	0.41	0.01	34.80	2E-265	1.74
CVD	Stroke	0.23	0.01	19.47	2E-84	0.56
CVD-MDD	AF	0.19	0.01	27.44	1E-165	0.43
CVD-MDD	CAD	0.71	0.01	110.49	<5E-324	6.15
CVD-MDD	HF	0.49	0.01	48.37	<5E-324	2.21
CVD-MDD	MDD	0.35	0.01	44.24	<5E-324	1.36
CVD-MDD	PAD	0.48	0.01	35.71	3E-279	1.84
CVD-MDD	Stroke	0.30	0.01	21.89	3E-106	0.71
MDD	AF	0.18	0.02	10.37	4E-25	0.06
MDD	CAD	0.41	0.02	26.41	9E-154	0.34
MDD	HF	0.38	0.03	14.83	1E-49	0.21
MDD	MDD	1.54	0.02	76.59	<5E-324	4.13
MDD	PAD	0.51	0.03	15.22	3E-52	0.33
MDD	Stroke	0.25	0.03	7.32	2E-13	0.08

Table S12. ICD codes (ICD10) used to define CVD and MDD outcomes in UK-Biobank, with corresponding sample size. Note that individuals with missing health record data were defined as controls.

Outcome	ICD code	ICD definition	N cases	N controls
Coronary artery disease	I25	Chronic ischemic heart disease	46,490	482,511
Heart failure	I50	Heart failure	15,271	513,730
Atrial fibrillation	I48	Atrial fibrillation and flutter	33,570	495,431
Stroke	I63	Acute Ischemic Stroke	8,164	520,837
Peripheral artery disease	I73	Other peripheral vascular diseases	8,544	520,457
MDD	F32/ F33	Depressive episode/ major depressive disorder, recurrent	27,776	501,225

Table S13. Results from Genomic SEM for the association between MDD and CVD/ risk factors while adjusting for (groups of) risk factors

Excel sheet TableS13

Table S14. Comparison of the effect of MDD on CVD when risk factors are modeled as mediators versus covariates in Genomic SEM. When there is strong attenuation (non overlapping confidence intervals are bold faced) this supports for the interpretation that the risk factor mediates the relationship between MDD and CVD.
lCI = lower limit of 95% confidence interval; uCI = upper limit of 95% confidence interval

Trait	Mediation - lCI	Mediation - uCI	Covariation - lCI	Covariation - uCI	% attenuation
**Blood pressure**	0.220	0.259	0.247	0.284	10.69
Diastolic Blood Press.	0.213	0.280	0.219	0.288	2.94
Systolic Blood Pressure	0.212	0.278	0.219	0.287	2.90
Pulse Pressure	0.223	0.290	0.221	0.290	-0.90
**Psychosocial**	-0.091	0.182	0.210	0.276	143.25
Educational attainment	0.155	0.224	0.216	0.283	28.12
Smoking	0.142	0.221	0.215	0.281	33.83
Loneliness	0.147	0.269	0.220	0.289	32.94
Exercise	0.163	0.239	0.218	0.286	25.41
Physical activity	0.200	0.272	0.222	0.292	9.95
Sleep duration	0.222	0.293	0.221	0.290	-0.48
Childhood maltreatment	0.168	0.271	0.217	0.287	22.46
**Metabolic**	4.344	9.515	0.217	0.286	-1898.92
Triglycerides	0.178	0.250	0.228	0.299	22.11
High-density lipo.	0.187	0.255	0.223	0.292	16.20
Non-high-density lipo.	0.219	0.288	0.229	0.299	4.16
Type II diabetes	0.143	0.211	0.216	0.284	33.63
BMI	0.160	0.228	0.201	0.267	20.38
Total cholesterol	0.221	0.291	0.224	0.293	1.01
Low-density lipo.	0.222	0.291	0.225	0.294	1.18
**Inflammation**	0.081	0.193	0.210	0.277	61.39
C-reactive protein	0.164	0.234	0.211	0.279	22.44
IL6	0.147	0.252	0.218	0.287	32.46

Table S15. Full results for the Mendelian randomization sensitivity analysis (univariable), including the number of instruments (N SNPs), I-squared (I2), instrument strength F, heterogeneity Q, and MR Egger intercepts. At significant pleiotropy, (MR Egger intercept p-value<.05) all results for that trait combination should be interpreted with caution, and is printed in orange. When there is significant heterogeneity (Qpval<.05) this indicates that the SNP instruments did not have uniform effects (i.e., varying in strength or direction). Significant Q-pvalues are flagged. When I2 is insufficient (weighted and unweighted below .9) the MR Egger results should not be interpreted; this did not occur in our results. When instrument strength is insufficient (F<10) results should be interpreted with caution; this did not occur in our results.

Excel sheet TableS15

Table S16. Results from the multivariable MR with MDD as exposure, the CVDs as outcome, and (grouped) risk factors as mediators.

Excel sheet TableS16

Table S17. Full results for the Mendelian randomization sensitivity analysis (univariable), including the number of instruments (N SNPs), I-squared (I2), instrument strength F, heterogeneity Q, and MR Egger intercepts. At significant pleiotropy, (MR Egger intercept p-value<.05) all results for that trait combination should be interpreted with caution, and is printed in orange. When there is significant heterogeneity (Qpval<.05) this indicates that the SNP instruments did not have uniform effects (i.e., varying in strength or direction). Significant Q-pvalues are flagged. When I2 is insufficient (weighted and unweighted below .9) the MR Egger results should not be interpreted; this did not occur in our results. When instrument strenght is insufficient (F<10) results should be interpreted with caution; this did not occur in our results.

Excel sheet TableS17
